# Rolipram plays an anti-fibrotic effect in ligamentum flavum fibroblasts by inhibiting the activation of ERK1/2

**DOI:** 10.1186/s12891-021-04712-9

**Published:** 2021-09-23

**Authors:** Likang Wu, Lei Xu, Yu Chen, Guohua Xu, Qunfeng Guo, Depeng Meng, Jianping Fan, Guoqiang Song, Peng Xu

**Affiliations:** 1 Department of Orthopedics, Changzheng Hospital, Naval Medical University, Shanghai, 200003 People’s Republic of China; 2 School of Pharmacy & School of Medicine, ChangZhou University, Changzhou, 213164 People’s Republic of China; 3 Institute of Bioinformatics and Medical Engineering, School of Electrical and Information Engineering, Jiangsu University of Technology, Changzhou, 213001 People’s Republic of China; 4 Department of Orthopedics, First Clinical Medical College, Shandong University of Traditional Chinese Medicine, Jinan, 250355 People’s Republic of China; 5 Institute of Bioinformatics and Medical Engineering, School of Electrical and Information Engineering, Jiangsu University of Technology, Changzhou, 213001 People’s Republic of China

**Keywords:** Rolipram, Phosphodiesterase, Ligamentum Flavum hypertrophy, ERK1/2, Fibrosis, TGF-β1

## Abstract

**Background:**

Fibrosis is an important factor and process of ligamentum flavum hypertrophy. The expression of phosphodiesterase family (PDE) is related to inflammation and fibrosis. This article studied the expression of PDE in hypertrophic ligamentum flavum fibroblasts and investigated whether inhibition of PDE4 activity can play an anti-fibrotic effect.

**Methods:**

Samples of clinical hypertrophic ligamentum flavum were collected and patients with lumbar disc herniations as a control group. The collagenase digestion method is used to separate fibroblasts. qPCR is used to detect the expression of PDE subtypes, type I collagen (Col I), type III collagen (Col III), fibronectin (FN1) and transforming growth factor β1 (TGF-β1). Recombinant TGF-β1 was used to stimulate fibroblasts to make a fibrotic cell model and treated with Rolipram. The morphology of the cells treated with drugs was observed by Sirius Red staining. Scratch the cells to observe their migration and proliferation. WB detects the expression of the above-mentioned multiple fibrotic proteins after drug treatment. Finally, combined with a variety of signaling pathway drugs, the signaling mechanism was studied.

**Results:**

Multiple PDE subtypes were expressed in ligamentum flavum fibroblasts. The expression of PDE4A and 4B was significantly up-regulated in the hypertrophic group. Using Rolipram to inhibit PDE4 activity, the expression of Col I and TGF-β1 in the hypertrophic group was inhibited. Col I recovered to the level of the control group. TGF-β1 was significantly inhibited, which was lower than the control group. Recombinant TGF-β1 stimulated fibroblasts to increase the expression of Col I/III, FN1 and TGF-β1, which was blocked by Rolipram. Rolipram restored the increased expression of p-ERK1/2 stimulated by TGF-β1.

**Conclusion:**

The expressions of PDE4A and 4B in the hypertrophic ligamentum flavum are increased, suggesting that it is related to the hypertrophy of the ligamentum flavum. Rolipram has a good anti-fibrosis effect after inhibiting the activity of PDE4. This is related to blocking the function of TGF-β1, specifically by restoring normal ERK1/2 signal.

**Supplementary Information:**

The online version contains supplementary material available at 10.1186/s12891-021-04712-9.

## Introduction

Hypertrophy of the ligamentum flavum (LF) is an important factor in lumbar spinal stenosis, causing pain and difficulty walking [1]. Surgery is currently the main treatment method, but it is traumatic and difficult [2]. People hope to explore the causes of LF hypertrophy and find safe and efficient treatment strategies. In the process of LF hypertrophy, abnormal mechanical stress stimulates chronic inflammation and changes in the tissue microenvironment [3]. Infiltrating macrophages secrete a large number of inflammatory factors and activate fibroblasts [4]. Stimulates the secretion and deposition of collagen fibers, which eventually causes the LF hypertrophy [5]. Therefore, inflammation and fibrosis are two important factors for LF hypertrophy [6]. Substances that exert anti-inflammatory and anti-fibrotic effects may be beneficial to the treatment of LF hypertrophy.

Phosphodiesterase (PDE) is widely expressed in organisms. Changes in its expression and activity are related to inflammation and fibrosis [7]. In multiple reports, inhibiting PDE activity has played an anti-fibrotic effect. For example, PDE4 inhibitors can antagonize fibroblast activation, thereby improving renal fibrosis [8] and inhibit fibroblast-mediated contraction of three-dimensional collagen gels in lung fibrosis [9]. In this study, a number of clinical samples of the LF were collected to detect differences in the expression of PDE subtypes in the two fibroblasts. Explore the relationship between the expression of PDE subtypes and LF hypertrophy. Then, we conducted further research on the increased expression of PDE4A and 4B. The specific inhibitor rolipram was used to inhibit the activity of PDE4A and 4B to investigate the anti-fibrosis effect.

## Materials and methods

### Specimens

This study was approved by Committee on Ethics of Biomedicine, Second Military Medical University (Shanghai, China). Lumbar spine 4/5 segment LF samples were collected from 25 patients who underwent lumbar surgery at Shanghai Changzheng Hospital from June 2019 to December 2019. A total of 15 patients with definite LF hypertrophy constituted the hypertrophy group (LFH) and 10 patients with lumbar disc herniation or trauma constituted the control group (NLFH). LF thickness was measured at the facet joint level by T1-weighted magnetic resonance (MR) imaging.

### Compounds and drugs

Rolipram (CAS: 61413–54-5, CSNpharm, Shanghai, China), WAY-262611 (CAS: 1123231–07-1, CSNpharm), LY294002 (CAS: 154447–36-6, CSNpharm), SCH772984 (CAS: 942183–80-4, CSNpharm) were dissolved in DMSO. It was diluted 1000–10,000 times with complete medium before use and the final concentration in media was 0.01–1 μM. Rh-TGF-β1 (CST, #8915, USA) was dissolved in 20 mM citrate with a pH =3.0 according instructions provided by the manufacturer. The final concentration was 5 ng/mL.

### LF fibroblast isolation and culture

LF tissue samples harvested from lumbar spinal stenosis patients were washed in physiological saline, minced and incubated for 1 h at 37 °C in Dulbecco’s Modified Eagle Medium (DMEM; HyClone, USA) containing 0.2% type I collagenase (Sigma, USA). The suspension was filtered using a 70 mm-mesh cell strainer (Falcon, BD, USA) and cells were seeded into the wells of a 60 mm culture dish (NEST, Wuxi, China) containing DMEM supplemented with 10% fetal bovine serum (FBS; Transgen, Beijing, China), 100 U/mL penicillin and 100 pg/mL streptomycin (HyClone). Subsequent experiments were performed using cells that were passaged between 2 and 5 times.

### RNA isolation and quantitative RT-PCR (qRT-PCR)

Total RNA was isolated from samples or cells using the Total RNA Isolation Kit (Vazyme, Nanjing, China). Next, cDNA was reverse transcribed from isolated RNA by incubating 1 μg of DNase treated RNA with components of a first-strand synthesis kit (Promega, USA). Real-time PCR was performed using TB Green® Premix Ex Taq™ (TAKARA, Japan) in a thermal cycler with the following parameters: 40 cycles, 95 °C for 10 s, 60 °C for 30 s. All primers (Table 1) were synthesized by Qingke Inc. (Shanghai, China). Data were analyzed using the 2^^-ΔΔCT^ method to compare gene expression levels.

**Table 1:** Forward and reverse primer sequences for LF hypertrophy markers and PDE isoforms used in qRT-PCR

Gene	Forward primer	Reverse prime
PDE1A	TGAAGGGATTGACAGAGC	ATGGTCCACCGATAATGC
PDE1B	CGCCGAGCAGAGGAGAAG	AGAACCGCAGTAGAGTAAGTGG
PDE1C	TGGAAGTGGGATACAGCAAGC	CTCCGTCAGCCAGTTCGC
PDE2A	ATCTTTGCCTTGTTTATTTCCTG	CAGCCAGCACAGATTTCG
PDE3A	GATGATAAATACGGATGTCTGTC	ACCGCCTGAGGAGCACTAG
PDE3B	TGCCTTCTTCTTCCTCACCTG	GACCACCACTGCCACACC
PDE4A	TTCACGGACCTGGAGATTC	TGAGGAACTGGTTGGAGAC
PDE4B	CAAGCCTAAACAATACAAGCATC	TGAGAATATCCAGCCACATTAAAG
PDE4C	CACCTGGCTGTGGGCTTC	ACTCAGTCGCTGCTTGGC
PDE4D	CTACTGGCTGATTTGAAGACTATG	GCTGGAGAGGCTTTGTTGG
PDE5A	ATCAGGAAACGGTGGGACATTTAC	CTTGTTCTCCAGCAGTGAAGTCTC
PDE7A	AGATAGGTGCTCTGATACTAG	ATGTCTGTGTCTGGTGTC
PDE7B	GGCTTCTTGCTCATTTGC	CCTGTTGATGTCTGTTGC
PDE8A	ATGTTTGCTCGCTTTGGAATC	CAGAATGTGTAGAATTGTGGTAGG
PDE8B	CAAATCCCTCCGAGCACAC	CTCCATAAATCTCCTGTTGAAGC
PDE9A	CGTGGAATTGGAAGGACTAAAAG	GAGTCAACTTCTTGTGGTTATCC
PDE10A	AACTATCGGCGGGTTCCTTATC	GCGTGTGATTGTTCTGAAGTATGG
PDE11A	CTGCTGGGTTTCAAGACATTC	GCTTGGAAGGCATTGTTGG
TGF-β1	CTGTACATTGACTTCCGCAAG	TGTCCAGGCTCCAAATGTAG
Col I	AAAGATGGACTCAACGGTCTC	CATCGTGAGCCTTCTCTTGAG
Col III	CGCCCTCCTAATGGTCAAGG	TTCTGAGGACCAGTAGGGCA
β-actin	GCGGGAAATCGTGCGTGACA	GGAAGGAAGGCTGGAAGAGTGC

### Western blot analysis

Tissue specimens and cells were lysed using RIPA buffer containing a protease inhibitors cocktail (Epizyme, Shanghai, China). Lysates were heated in 95 °C for 10 min in protein sample loading buffer (Epizyme). Total cell lysates were separated using 10% SDS-PAGE (Epizyme) and transferred to an Immobilon-P Transfer Membrane (Millipore, USA). Membranes were blocked using 5% nonfat milk dissolved in Tris-buffered saline and then incubated with a FN1 (CST, #26836, USA), ERK1/2 (CST, #4695, USA), p-ERK1/2 (CST, #4370, USA), TGF-β (CST, #3711, USA), COL3A1 (CST, #30565, USA), collagen I (Abcam, ab34170, USA) and α-SMA (Servicebio, GB11044, China) antibodies. The following day, membranes were incubated with a horseradish peroxidase-conjugated secondary antibody (Transgen). GAPDH (Transgen) and β-tubulin (Transgen) were used as loading controls. Finally, membranes were visualized using an ECL western blotting detection reagent (Epizyme).

### Statistics and heat map drawing

Statistical analyses and graphs were performed and generated using GraphPad Prism 8 Software. Differences in the fold change between patients and controls were assessed using an independent-samples t test. A *P* value less than 0.05 was considered as statistically significant. Use GraphPad software to draw the heat map. The shade of the color in the figure represents the △△Ct value of the qPCR result.

## Results

### The expression of PDE subtypes in the LF fibroblast

**Fig. 1:**
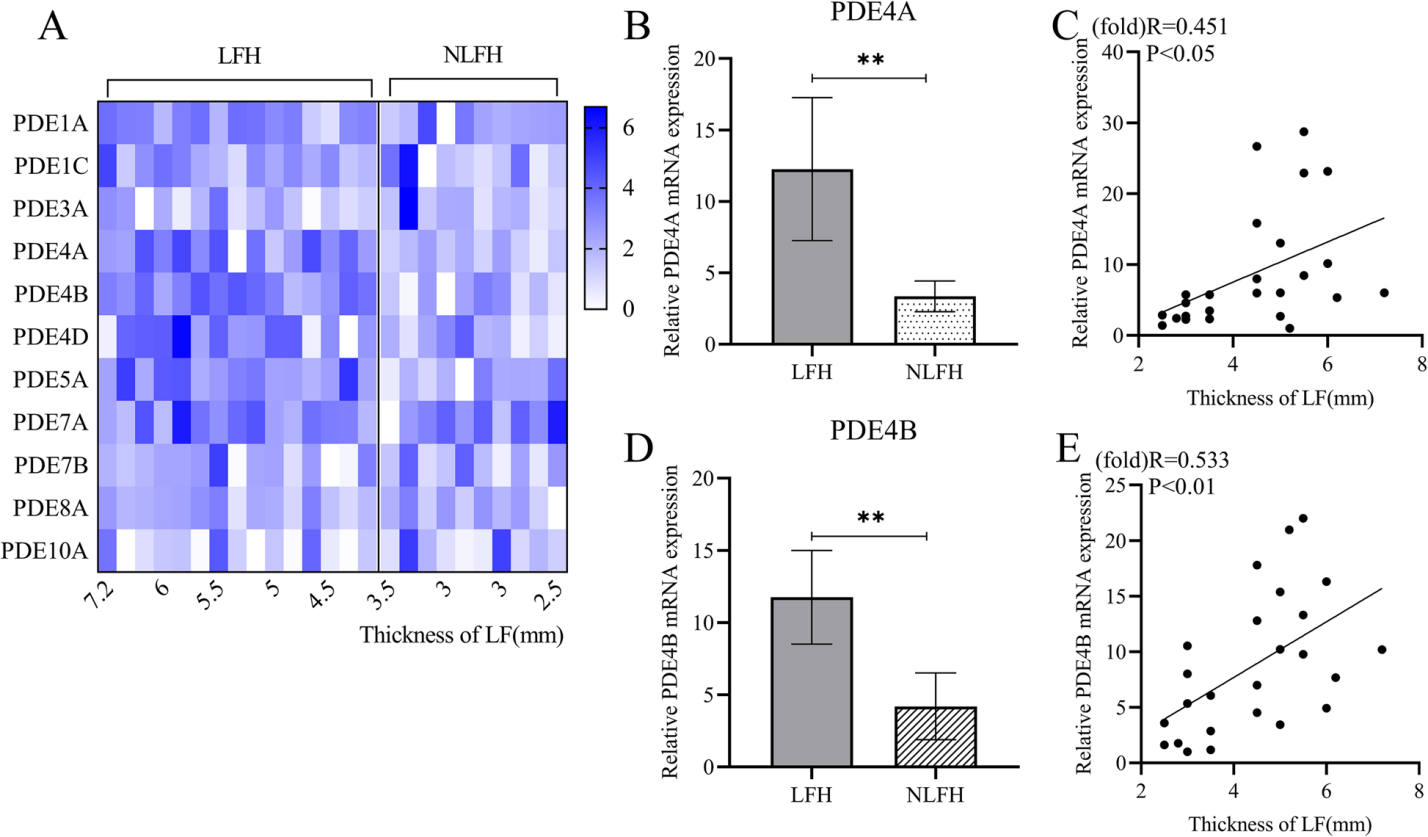
The expression of PDE4A and 4B in hypertrophic fibroblasts is up-regulated. **A** Heatmap of differentially expressed PDEs. Data shown is mRNA expression levels of LF samples using qRT-PCR. The shade of the color in the figure represents the _△△_Ct value of the qPCR result. LFH stands for LF hypertrophy group and NLFH represents the control group. **B-E** qRT-PCR analysis of PDE4A and PDE4B mRNA expression levels in the LFH versus the NLFH groups. Data are presented as the mean ± 95%CI. **p < 0.01. The mRNA expression of PDE4A and PDE4B were related to the thickness of LF. The correlation coefficient (R) and probability (P) value obtained by regression analysis are shown in the figure

After extracting total RNA from 25 samples, a qPCR reaction was performed and the results were statistically summarized. Hypertrophic group and control constituted the expression of PDE family in fibroblasts (Fig. 1A). Among the investigated PDE subtypes, PDE1B, 2A, 3B, 4C, 7B, 8B, 9A and 11A are expressed in very little or almost no expression in LF fibroblasts. The expression levels of PDE4A and PDE4B in the hypertrophic group (LFH) were significantly higher than those in the control group (NLFH) (*P* < 0.01, Fig. 1B, D). The expression levels of other PDE subtypes also vary among individuals, but there is no statistical difference (Table 2). In addition, the expression of PDE4A and PDE4B increased as the thickness of LF increased (Fig. 1C, E).

**Table 2:** Correlation between gene in Fig. 1A mRNA expression and LF thickness

Gene name	R value	*P* value
PDE1A	0.2696	0.1924
PDE1C	0.05952	0.7775
PDE3A	−0.1015	0.6292
PDE4A	0.4510	0.0237*
PDE4B	0.5335	0.006**
PDE4D	0.3137	0.1268
PDE5A	0.3074	0.1349
PDE7A	−0.1082	0.6066
PDE7B	0.03061	0.8845
PDE8A	0.1679	0.4225
PDE10A	−0.08561	0.6841
TGF-β1	0.4484	0.0246*
Col I	0.6745	0.0002***
Col III	0.5051	0.01*

### The relationship between the up-regulation of PDE4A and B and fibrosis markers

**Fig. 2:**
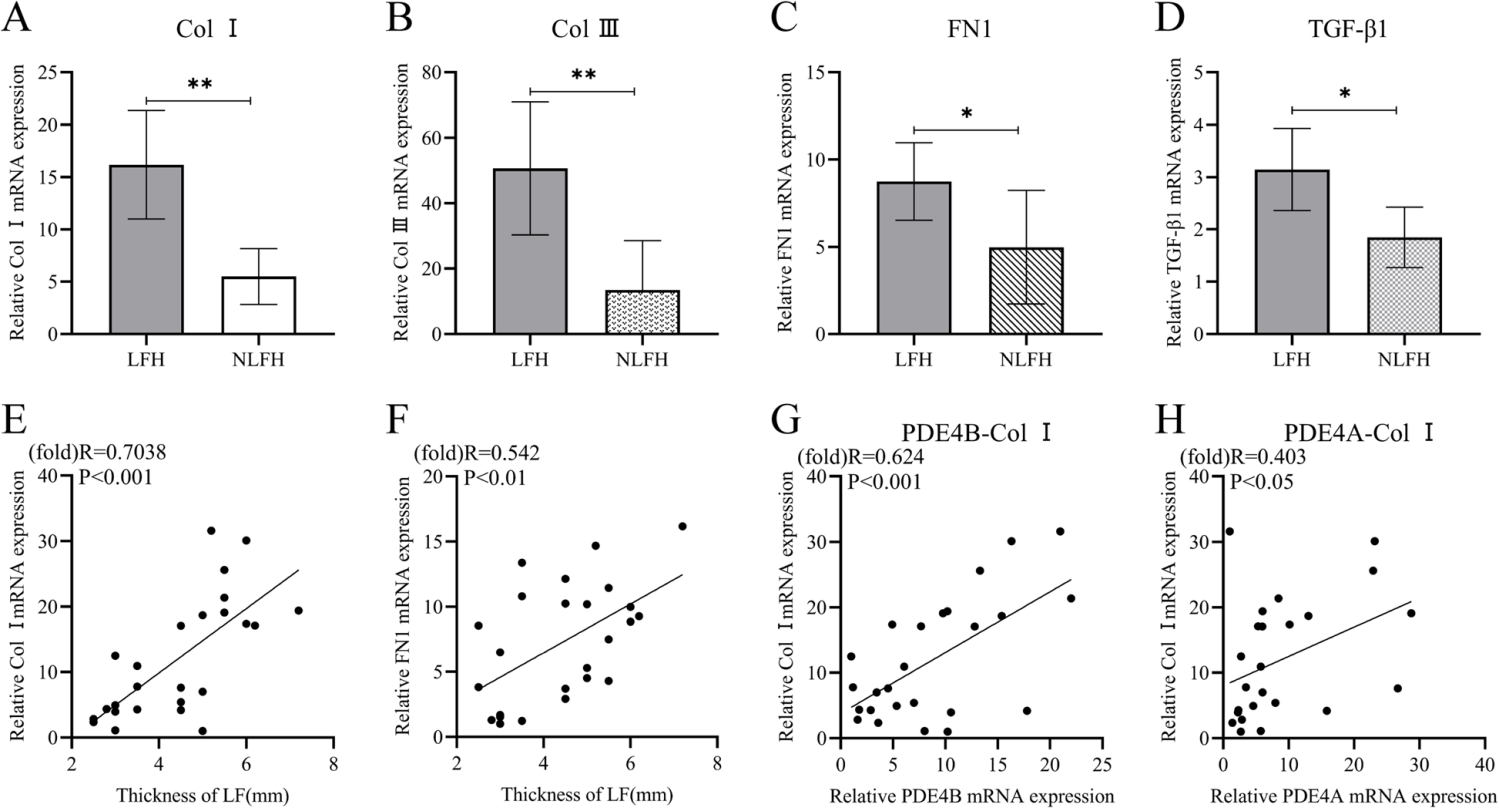
The expression of PDE4A and 4B is related to fibrosis. The histogram results in **A**, **B**, **C** and **D** were presented as the mean ± 95%CI. *p < 0.05, **p < 0.01. **E** The Col I mRNA expression was related to the thickness of LF. **F** There is a linear correlation between FN1 mRNA expression and LF thickness. **G**-**H** The mRNA expression of PDE4A and PDE4B were related to the Col I

We also tested the expression of markers related to LF hypertrophy. The expressions of Col I, Col III, FN1 and TGF-β1 were up-regulated in the hypertrophy group (Fig. 2A-D). The mRNA expression of Col I, FN1 has a linear correlation with the thickness of the LF (Fig. 2E, F). Comparing the relationship between the expression of PDE4A and 4B and the expression of Col I, the expression of PDE4B and Col I has a linear correlation (R = 0.5947, *P* < 0.01, Fig. 2G). The linear correlation between the expression of PDE4A and Col1 is shown in the Fig. 2H.

### Rolipram inhibits the expression of col I and TGF-β1 in hypertrophic fibroblasts

**Fig. 3:**
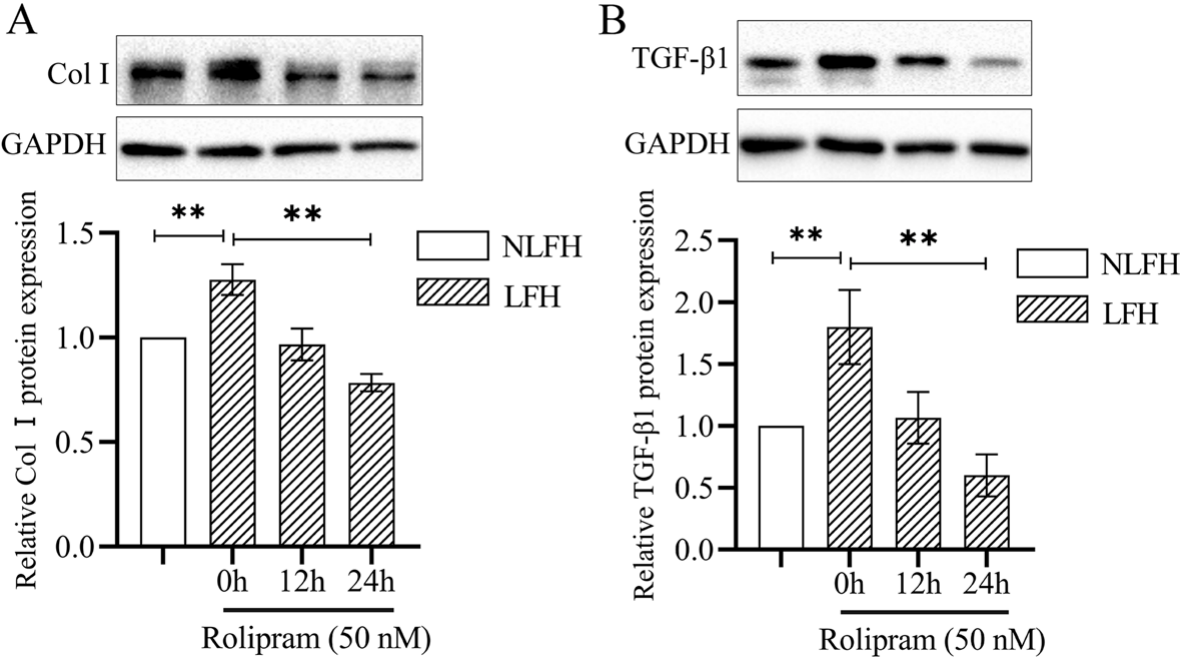
Inhibiting PDE4 with Rolipram attenuates Col I and TGF-β1 expression levels in LF hypertrophy fibroblasts. Primary LF fibroblasts were treated with rolipram (50 nM) at different times. Western blot analysis shows the expression for Col I and TGF-β1 in LF fibroblasts treated with Rolipram. GAPDH served as an endogenous control. **P < 0.01

Rolipram can inhibit the activity of all PDE4 subtypes. Compared with normal LF fibroblasts, the expression of Col I and TGF-β1 increased in the hypertrophic group (Fig. 3A, B). After Rolipram inhibited the activity of PDE4 in hypertrophic LF fibroblasts, the expression levels of Col I and TGF-β1 decreased. The expression of TGF-β1 dropped to a very low level after Rolipram treatment, which was lower than that of the control group. The expression of Col I in the hypertrophy group was down-regulated after Rolipram treatment, which was tended to the expression level of Col I in the control group.

### Rolipram blocks fibroblast fibrosis stimulated by TGF-β1

**Fig. 4:**
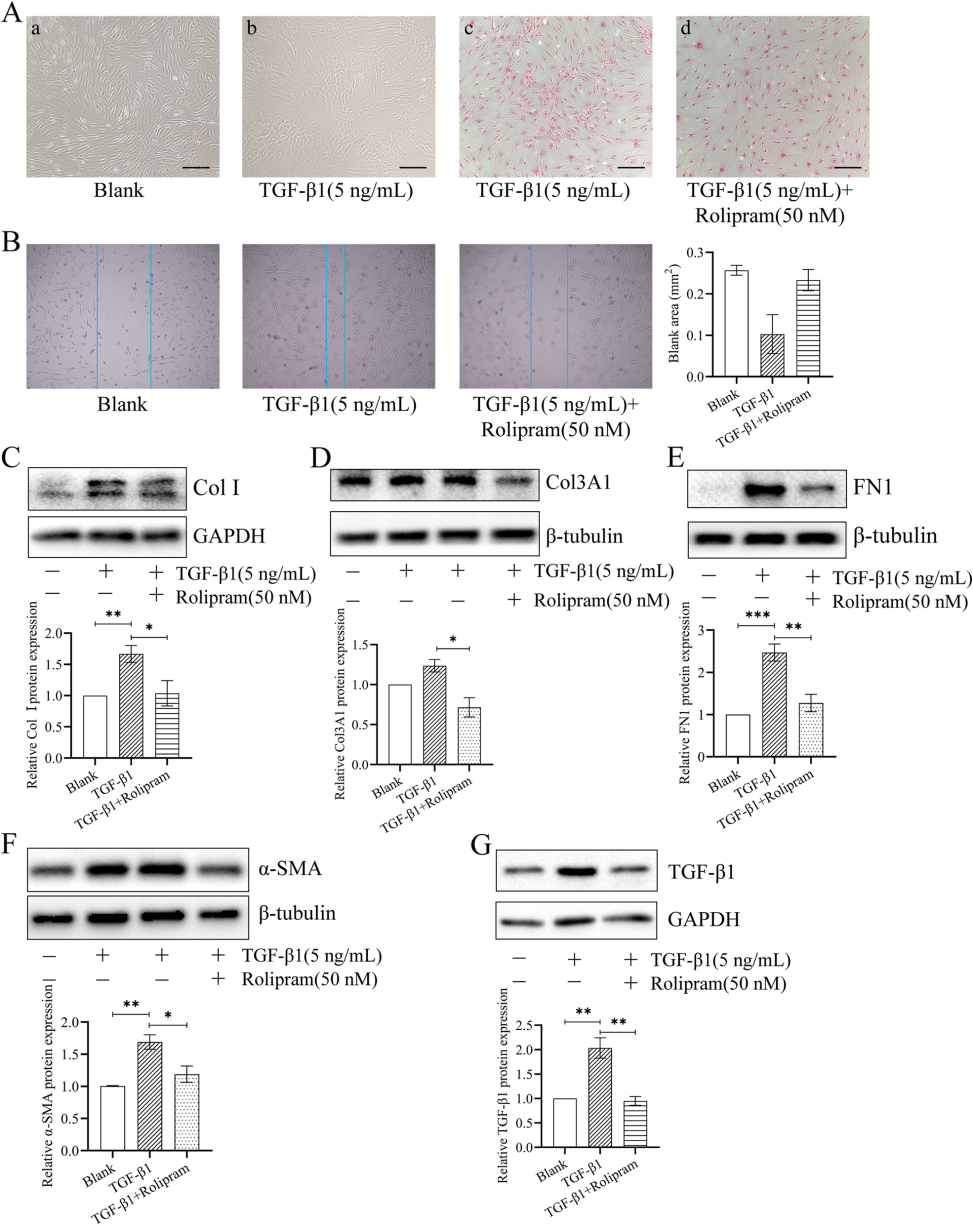
Rolipram blocks TGF-β1-induced LF fibroblast fibrosis. (A) a and b, Picture of fibroblasts (40x). c and d, staining with Sirius Red makes the fibroblasts easy to observe (100x). (B) Cell scratch test in LF fibroblasts (100×). (C-G) After fibroblasts were stimulated by TGF-β1 and treated with Rolipram, the expression of Col I, Col3A1, FN1, α-SMA and TGF-β1. GAPDH or β-tubulin served as an endogenous loading control. *p < 0.05, **p < 0.01, ***p < 0.001

Fibroblasts have enhanced proliferation and migration ability after stimulation by recombinant human TGF-β1. The morphology of the cell changes, from a spindle shape to a polygonal shape. The edges of the cells are blurred (Fig. 4A, B). The expression of fibrosis markers such as Col I, Col III, FN1 and α-SMA was up-regulated (Fig. 4C-F). Under the stimulation of exogenous TGF-β1, the expression of endogenous TGF-β1 in fibroblasts increases (Fig. 4G). After administration of Rolipram, the above-mentioned fibrotic protein expression was restored. The aggregation of fibroblasts was suppressed, but polygonal fibroblasts were still visible (Fig. 4A. d).

### The signaling pathway is involved in Rolipram’s anti-fibrosis effect

**Fig. 5:**
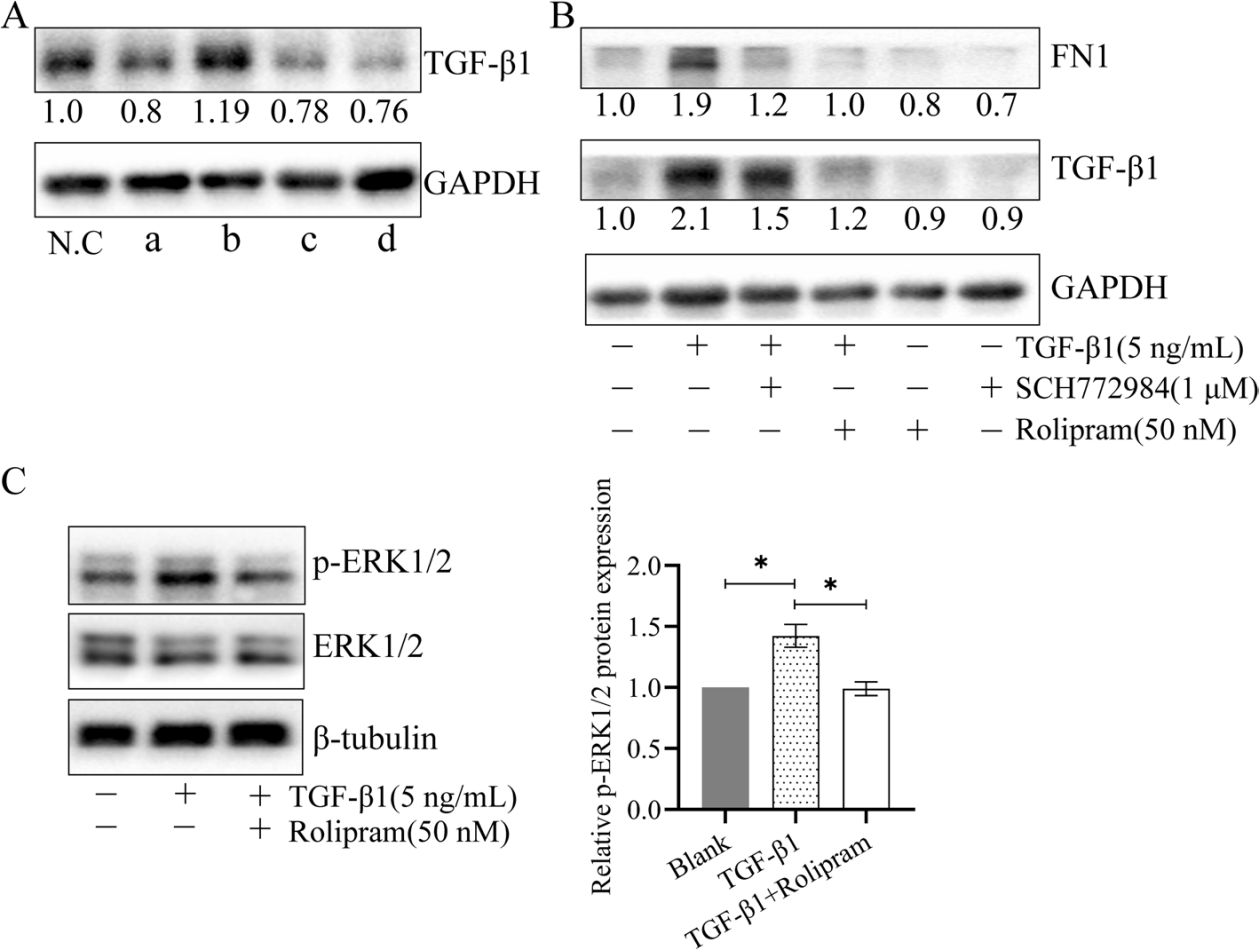
Rolipram restores ERK1/2 signaling to block TGF-β1 stimulated LF fibroblast fibrosis. (A) Different drugs were used to treat fibroblasts stimulated by TGF-β1. WB detects the expression of TGF-β1. a, Rolipram (50 nM) is a PDE4 inhibitor.; b, WAY-262611 (1 μM) is the Wnt/β-catenin pathway activator.; c, LY294002 (1 μM) is an inhibitor of the PI3K pathway; d, SCH772984 (1 μM) inhibits ERK1/2 signal transduction. (B) The expression of FN1 and TGF-β1 was up-regulated after exogenous TGF-β1 stimulation. Using Rolipram or SCH772984 blocked this change. (C) Western blot analysis reveals the expression levels of p-ERK1/2 and ERK1/2 in LF fibroblasts treated with TGF-β1 (5 ng/ mL) and Rolipram (50 nM, 24 h). β-tubulin served as an endogenous loading control. *p < 0.05

The fibroblasts stimulated by TGF-β1 were treated with four drugs and the expression of TGF-β1 in the fibroblasts was detected. The results showed that Rolipram reduced the expression of TGF-β1 (Fig. 5A. a). Activation of the β-catenin pathway increases the expression of TGF-β1 (Fig. 5A. b). Inhibiting the signal transduction of ERK1/2 and PI3K showed the effect of inhibiting the expression of TGF-β1 (Fig. 5A. c, d). We conduct in-depth research on the ERK1/2 pathway. The use of Rolipram and ERK1/2 inhibitor SCH772984 both reduced the expression of TGF-β1 and FN1 (Fig. 5B). After fibroblasts stimulated by TGF-β1, the expression of p-ERK1/2 increased, which was inhibited by Rolipram (Fig. 5C).

## Discussion

The dysregulation of PDE expression is related to many diseases [10]. We found that the mRNA expression of PDE4A and 4B was up-regulated in the hypertrophic group (LFH). The up-regulated PDE4A and 4B inactivate intracellular cAMP and cause a variety of signal pathway changes. In diethylnitrosamine-induced liver fibrosis, the overexpression of PDE4 is an important pathogenic factor. Enhancing cAMP and CREB levels by inhibiting PDE4 activity can regulate inflammation and fibrosis, thereby exerting a therapeutic effect [11]. In our results, the expression of PDE4B increased linearly with the expression of Col I. Col I is an important marker of fibrosis and LF hypertrophy [12]. This means that increased expression of PDE4B is related to fibrosis. Not only that, the expression of PDE4A and PDE4B increased linearly with the increase of the thickness of the LF. This implies that the increased expression of PDE4A and 4B is related to LF hypertrophy.

We used Rolipram, an inhibitor of PDE4A and 4B, to treat LF fibroblasts to investigate the effect of inhibiting PDE4 activity. In the hypertrophic LF, the expression of a variety of cytokines increases. Including TNF-α, IL-1β and IL-6 related to inflammation, TGF-β1, CTGF, Col I and Col III related to fibrosis [13]. After treating hypertrophic fibroblasts with Rolipram, the expression of Col I and endogenous TGF-β1 was down-regulated. And the expression of TGF-β1 was lower than that of the control group (Fig. 3). This indicates that Rolipram can significantly inhibit the expression of TGF-β1 in fibroblasts. Rolipram increases the content of cAMP in cells and stimulates the activation of downstream PKA and CREB, thereby inhibiting the function of TGF-β1 [14].

TGF-β1 is up-regulated in hypertrophic LF tissue and is one of the important factors leading to LF hypertrophy [15]. TGF-β1 antibodies and their inhibitors have been proven to be effective in treating certain fibrotic diseases by targeting the TGF-β1 signal transduction pathway [16]. For example, CCN5 protein inhibits the differentiation of fibroblasts induced by TGF-β1 into myofibroblasts, reducing fibrosis and LF hypertrophy [17]. Down-regulation of cytokine receptor-like factor 1 (CRLF1) reduces the fibrosis caused by inflammatory cytokines and mechanical stress by blocking the TGF-β1 pathway. In mouse models, knocking out CRLF1 can prevent the formation of LF hypertrophy [18]. In our experiment, Rolipram’s anti-fibrosis effect may be related to the blocking of TGF-β1 signal transduction, and we have conducted in-depth research on this.

There is a large gap between samples of primary fibroblasts. The expression of fibrotic proteins such as TGF-β1 and Col I is generally increased in the hypertrophic group, but there is a large gap in a specific single sample. TGF-β1 can promote the synthesis of extracellular matrix (ECM) protein and stimulate the proliferation and migration of fibroblasts [19]. Using recombinant TGF-β1 to stimulate fibroblasts to form a cell model of fibrosis, the expression of fibrotic protein is relatively stable, which is suitable for subsequent mechanism research. After stimulation by recombinant TGF-β1, the expression of a variety of fibrotic proteins increased, including Col I, Col III, FN1 and endogenous TGF-β1 (Fig. 4). FN1 plays an important role in the process of fibrosis and can affect the function of TGF-β1. In tissues, isolated TGF-β1 and ECM inhibit the activation of TGF-β1, thereby limiting its ability to stimulate cell surface receptors [20]. FN1 can also be used as a bridge to transport TGF-β1 and promote its function [21]. In addition, FN1 has increased expression in the hypertrophic LF. This means that the expression of FN1 is related to LF hypertrophy. After TGF-β1 stimulation, fibroblasts changed their morphology, increased their proliferation and migration capabilities (Fig. 4). There are similar results in the Hur’s report [22]. As an important marker of LF hypertrophy, TGF-β1 promotes fibrosis and causes tissue hypertrophy of the LF. The expression of these up-regulated fibrotic proteins was down-regulated after Rolipram treatment (Fig. 4). Rolipram exerts a certain anti-fibrosis effect.

The TGF-β family plays a key role in tissue fibrosis and mediates fibrosis through Smad-dependent or non-Smad pathways [23]. A variety of signaling pathways are related to TGF-β1 to stimulate fibrosis, including β-catenin, PI3K and ERK1/2 [24]. When drugs were used to interfere with the three signal transductions, the function of TGF-β1 changed to varying degrees (Fig. 5). We conducted a detailed study on ERK1/2 signal transduction. When TGF-β1 stimulates fibroblast activation, ERK1/2 is activated and the expression of p-ERK1/2 increases (Fig. 5). The activated ERK1/2 may affect the activity of PDE4 [10]. The up-regulated PDE4A and 4B deactivate cAMP hydrolysis and promote the process of fibrosis. The use of ERK1/2 pathway inhibitors or Rolipram can block the fibrosis stimulated by TGF-β1. Rolipram exerts an anti-fibrotic effect by restoring normal p-ERK1/2 expression.

In conclusion, we found that the expression of PDE4A and 4B mRNA in the hypertrophic LF is up-regulated, which is importantly related to the hypertrophy of the LF. Inhibiting the activity of PDE4A and 4B by Rolipram can inhibit a variety of fibrosis-related proteins and block the effect of TGF-β1 by restoring the expression of p-ERK1/2. This effect may be effective in treating LF hypertrophy.

## Supplementary Information

**Additional file 1.**

## Data Availability

The datasets used and/or analyzed during the current study are available from the corresponding author upon reasonable request.

## Abbreviations

LF: Ligamentum flavum
PDE: Phosphodiesterase
Col I: Collagen I
Col III: Collagen III
TGF-β1: Transforming growth factor β1
ERK1/2: Extracellular regulated protein kinases 1/2
FN1: Fibronectin
cAMP: Cyclic adenosine monophosphate
ECM: Extracellular matrix
CREB: cAMP-response element binding protein
CRLF1: Cytokine receptor-like factor 1
