# Supplementary figures and images for: Rolipram plays an anti-fibrotic effect in ligamentum flavum fibroblasts by inhibiting the activation of ERK1/2
Source: BMC Musculoskelet Disord. 2021 Sep 23;22:818. doi: 10.1186/s12891-021-04712-9 (PMC8461931; doi:10.1186/s12891-021-04712-9)

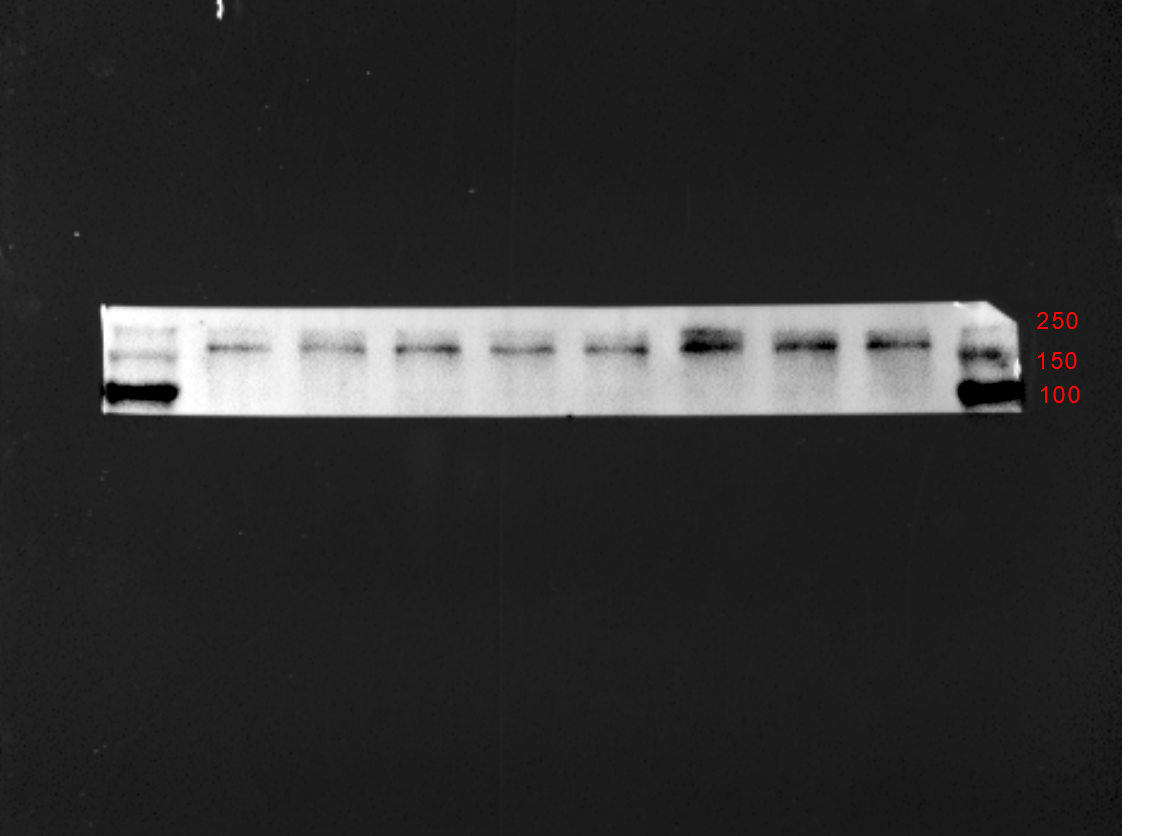

Supplement: Supplementary file 1 — Additional file 1. [file 12891_2021_4712_MOESM1_ESM.zip › merge/Fig. 3A-Col ó±.tif]

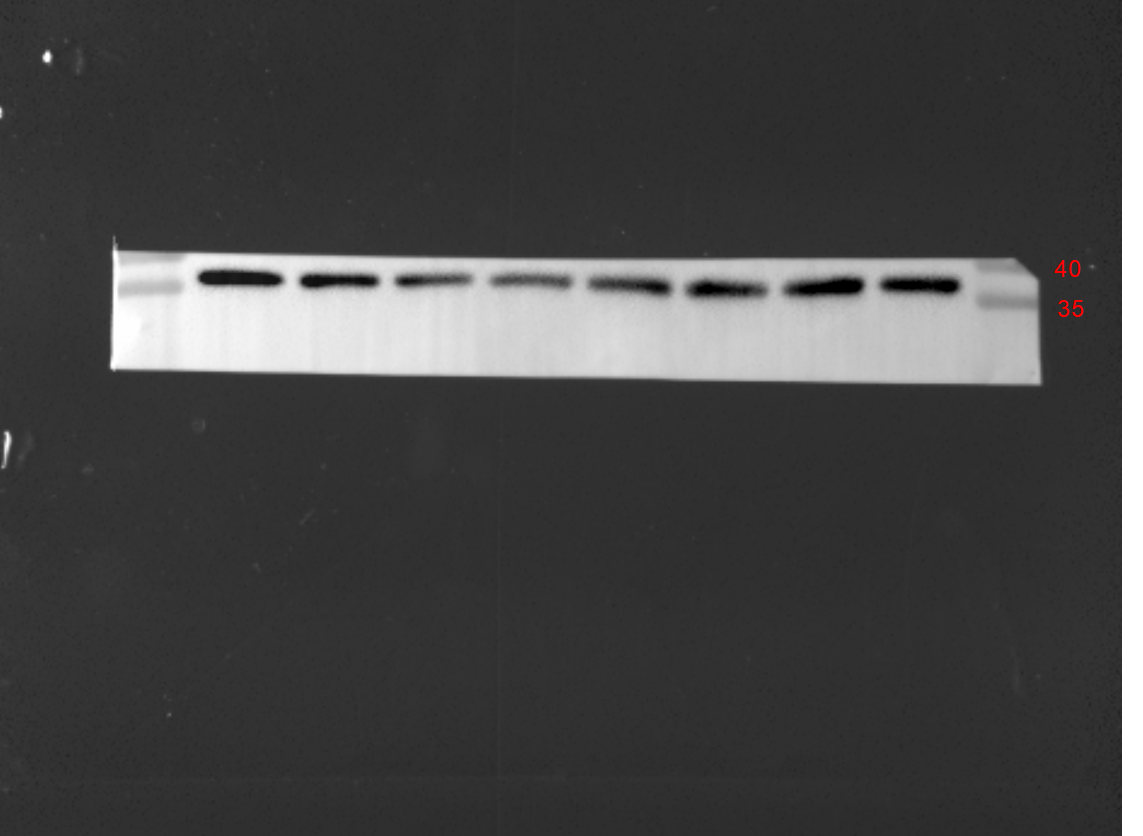

Supplement: Supplementary file 1 — Additional file 1. [file 12891_2021_4712_MOESM1_ESM.zip › merge/Fig. 3A-GAPDH.tif]

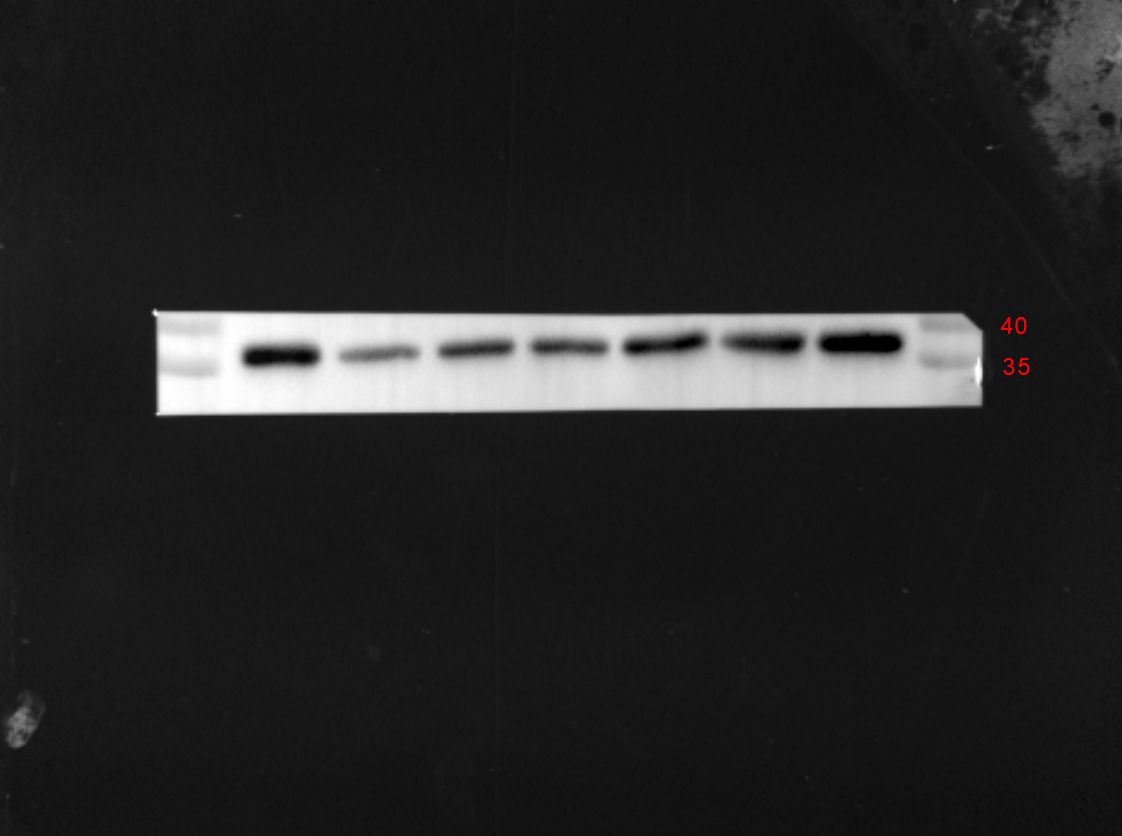

Supplement: Supplementary file 1 — Additional file 1. [file 12891_2021_4712_MOESM1_ESM.zip › merge/Fig. 3B-GAPDH.tif]

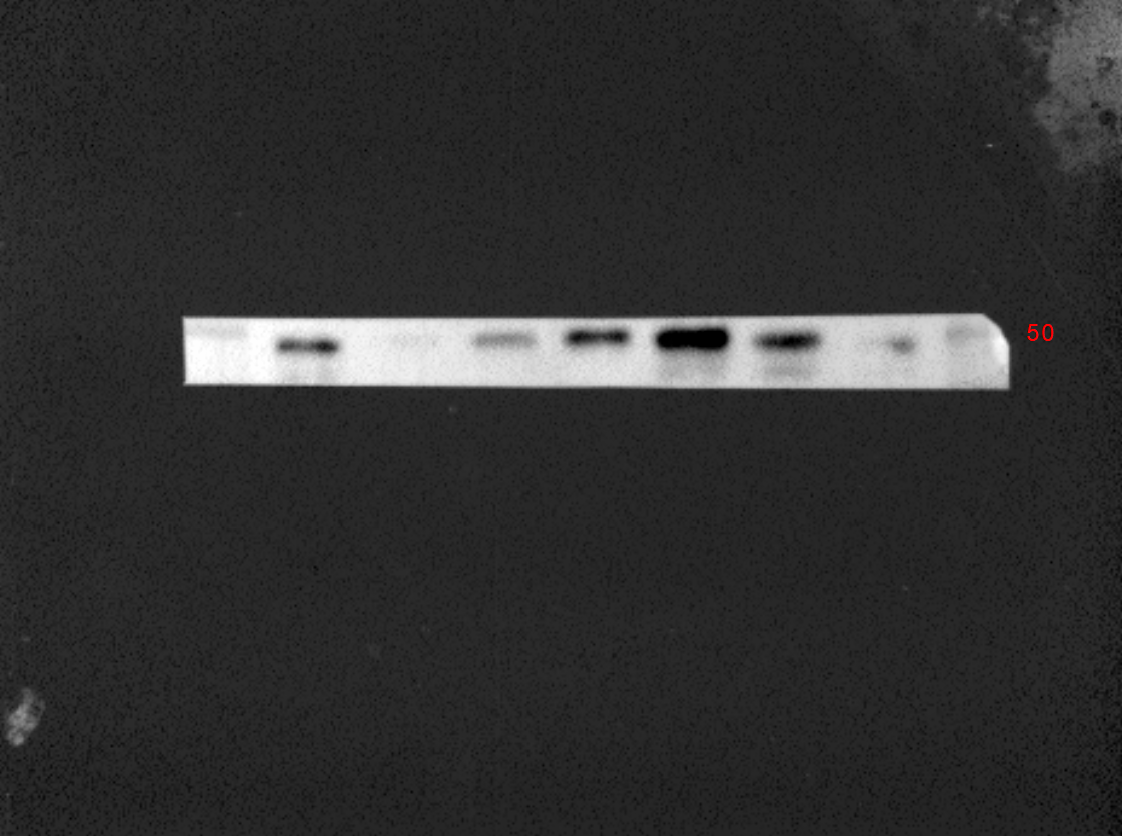

Supplement: Supplementary file 1 — Additional file 1. [file 12891_2021_4712_MOESM1_ESM.zip › merge/Fig. 3B-TGF-a┬1.tif]

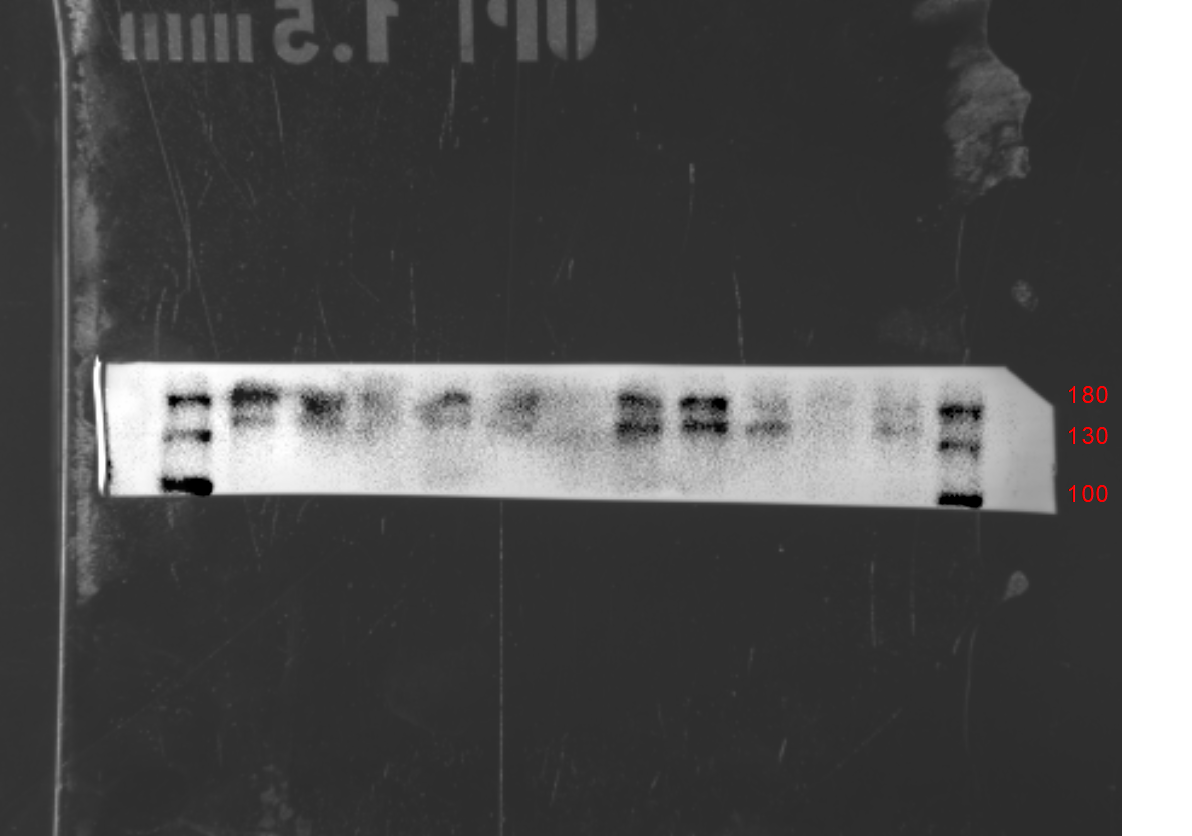

Supplement: Supplementary file 1 — Additional file 1. [file 12891_2021_4712_MOESM1_ESM.zip › merge/Fig. 4C-Col ó±.tif]

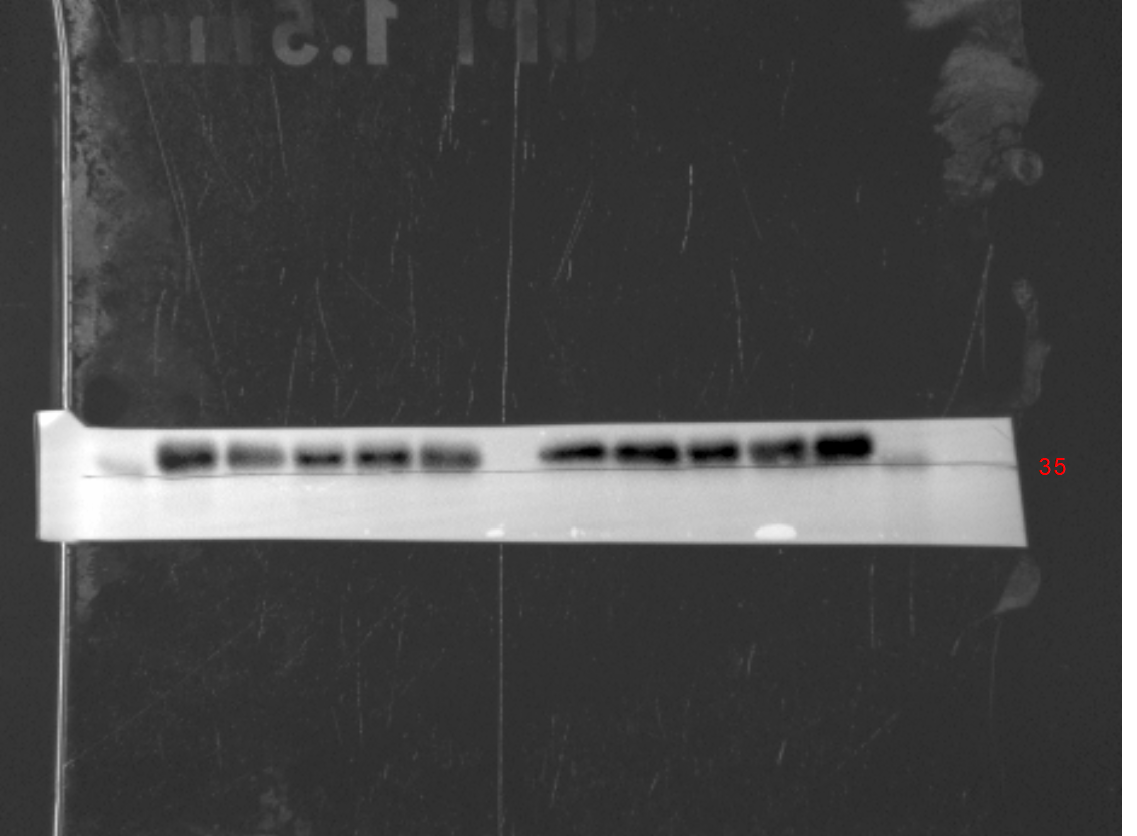

Supplement: Supplementary file 1 — Additional file 1. [file 12891_2021_4712_MOESM1_ESM.zip › merge/Fig. 4C-GAPDH.tif]

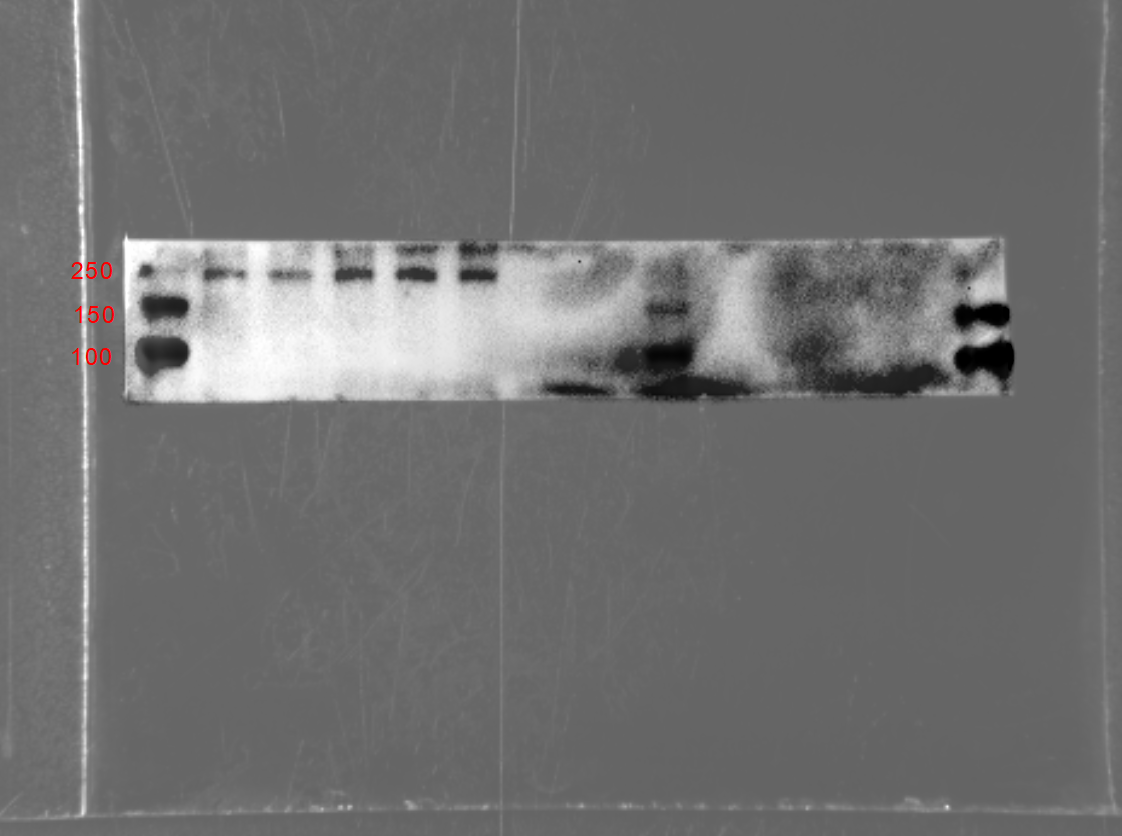

Supplement: Supplementary file 1 — Additional file 1. [file 12891_2021_4712_MOESM1_ESM.zip › merge/Fig. 4D-Col3A1.tif]

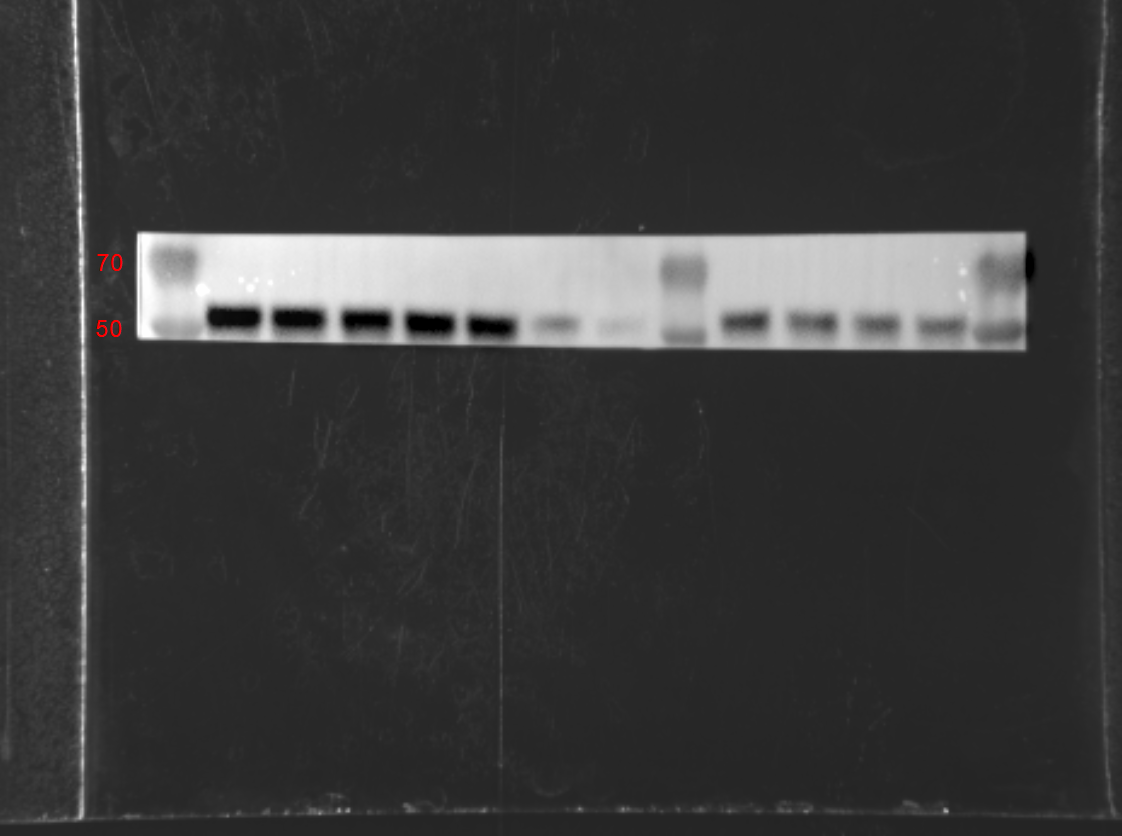

Supplement: Supplementary file 1 — Additional file 1. [file 12891_2021_4712_MOESM1_ESM.zip › merge/Fig. 4D-a┬-tubulin.tif]

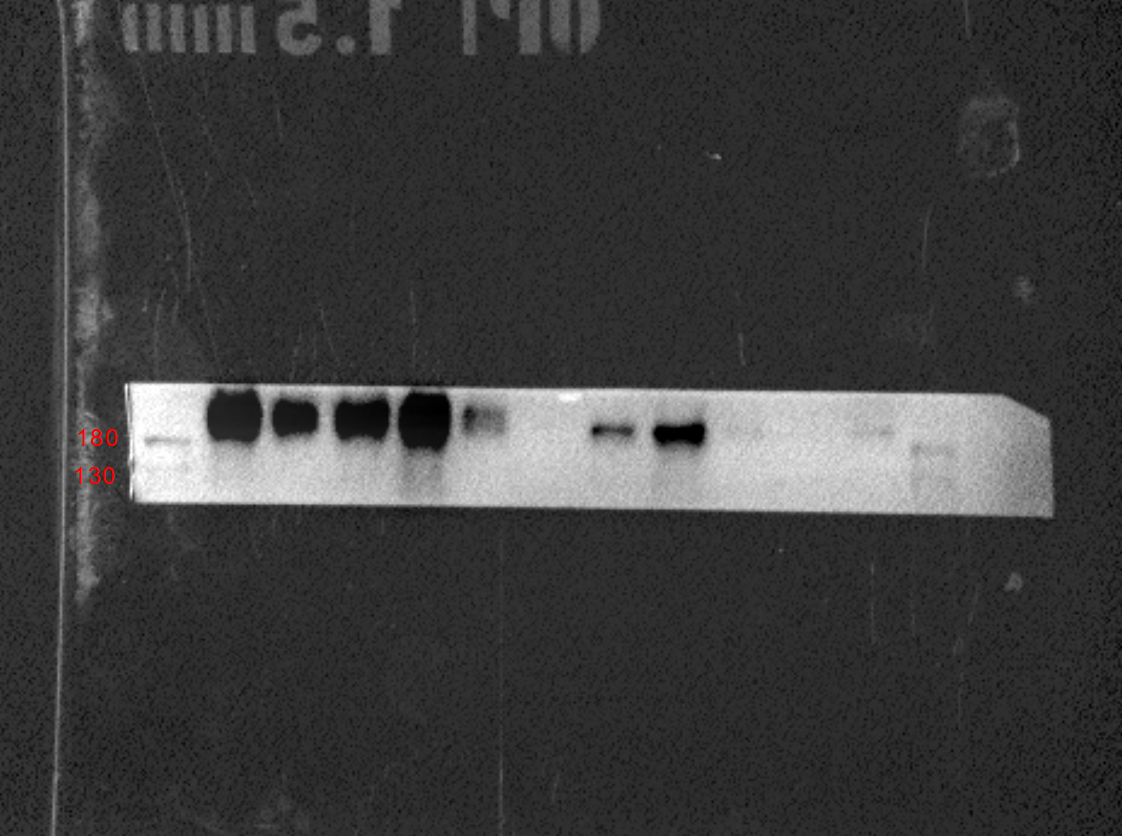

Supplement: Supplementary file 1 — Additional file 1. [file 12891_2021_4712_MOESM1_ESM.zip › merge/Fig. 4E-FN1.tif]

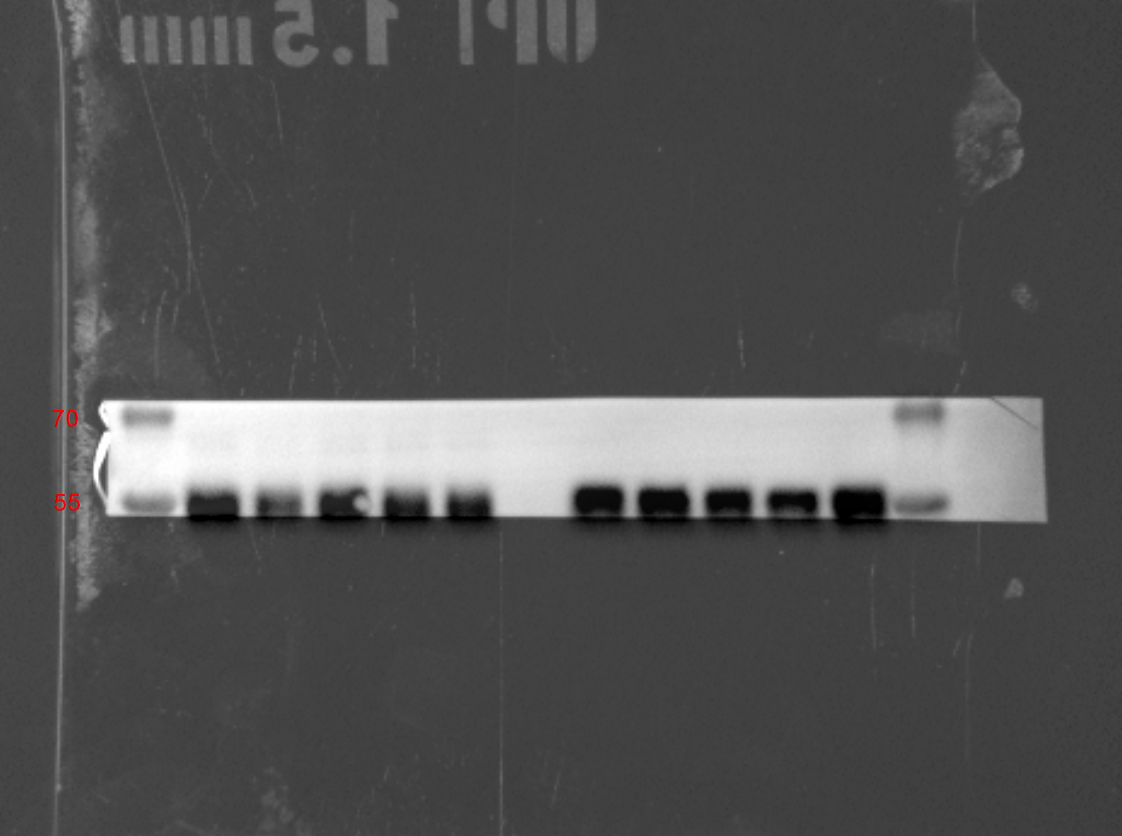

Supplement: Supplementary file 1 — Additional file 1. [file 12891_2021_4712_MOESM1_ESM.zip › merge/Fig. 4E-a┬-tubulin.tif]

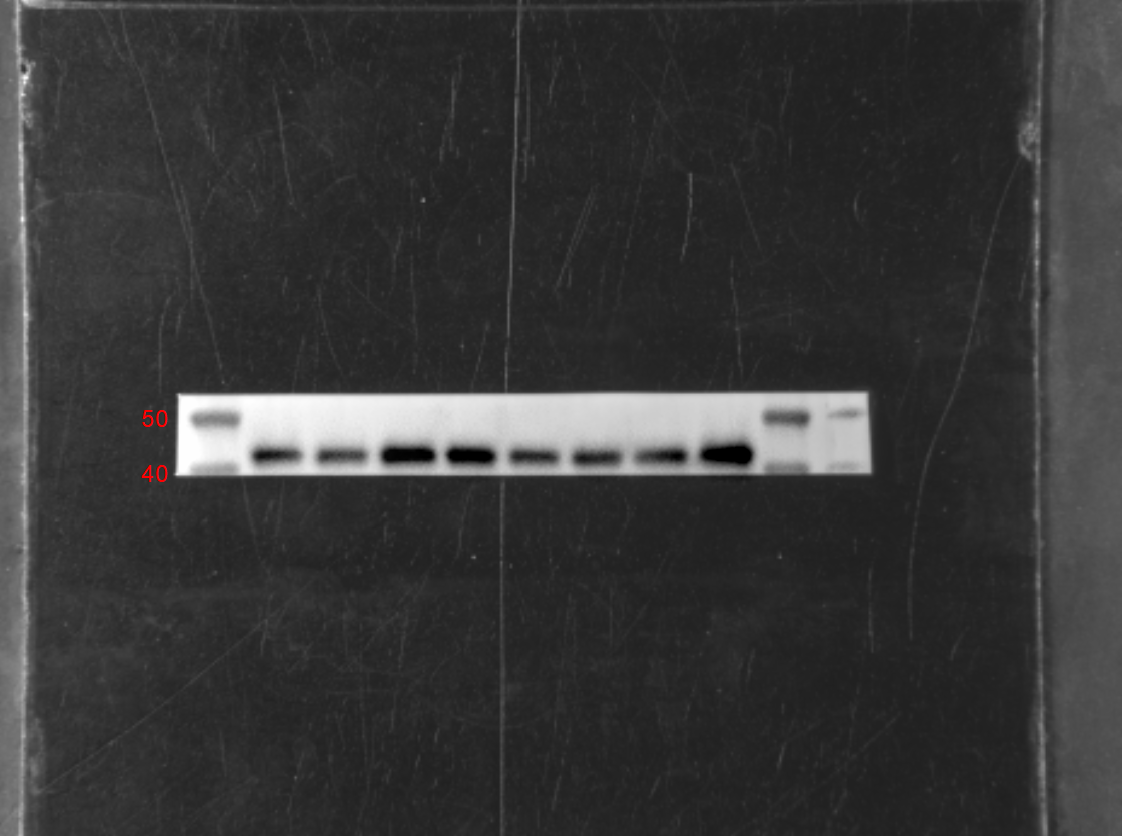

Supplement: Supplementary file 1 — Additional file 1. [file 12891_2021_4712_MOESM1_ESM.zip › merge/Fig. 4F-a┴-SMA.tif]

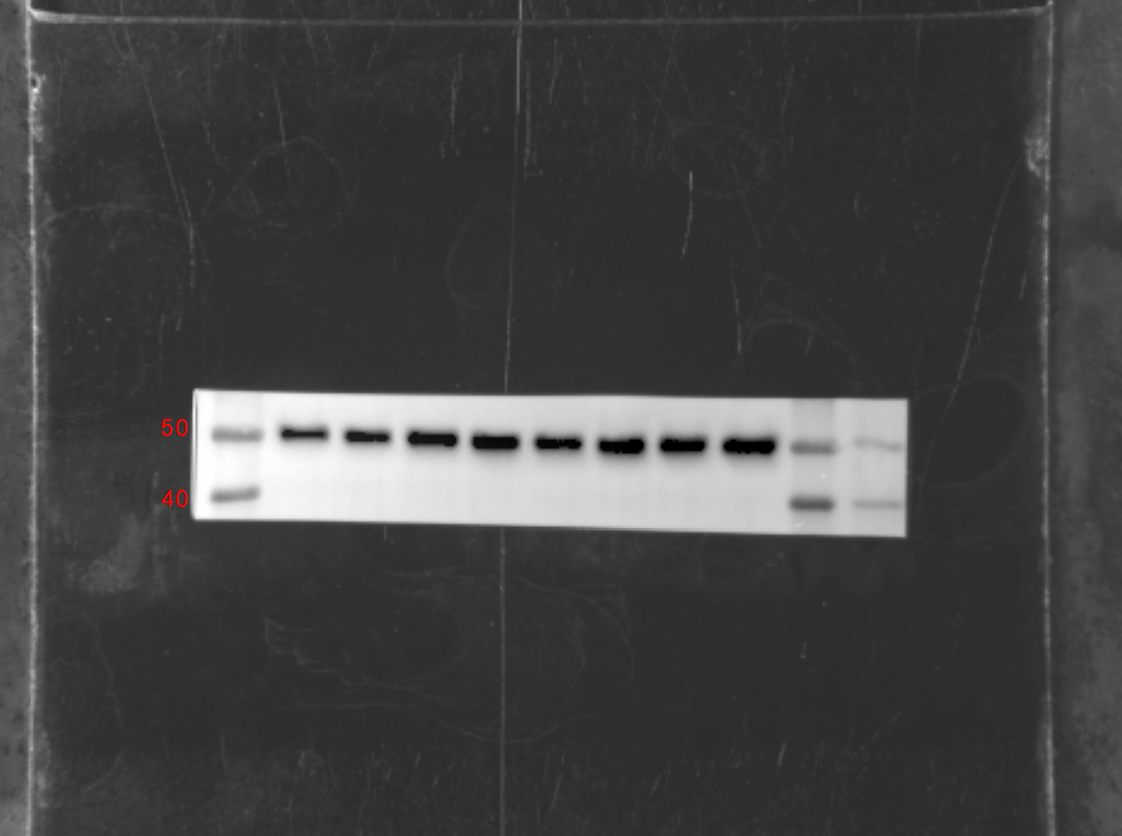

Supplement: Supplementary file 1 — Additional file 1. [file 12891_2021_4712_MOESM1_ESM.zip › merge/Fig. 4F-a┬-tubulin.tif]

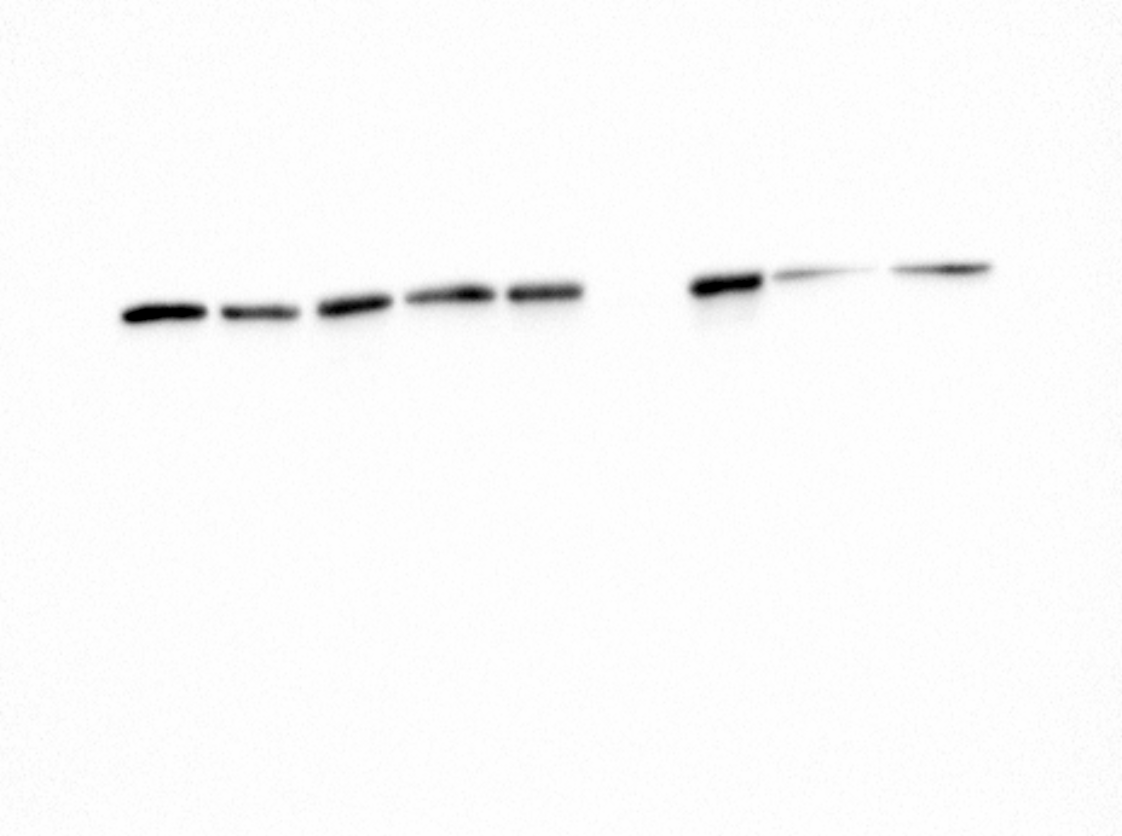

Supplement: Supplementary file 1 — Additional file 1. [file 12891_2021_4712_MOESM1_ESM.zip › merge/Fig. 4G-GAPDH.tif]

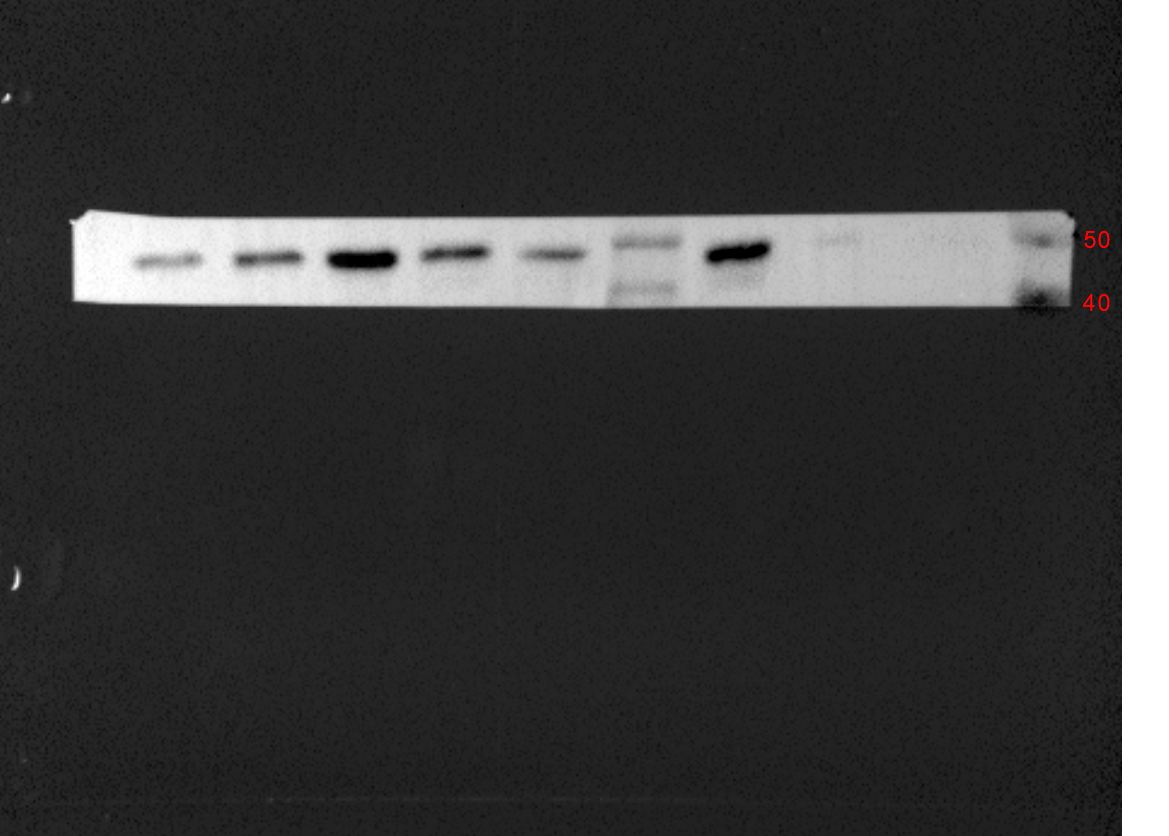

Supplement: Supplementary file 1 — Additional file 1. [file 12891_2021_4712_MOESM1_ESM.zip › merge/Fig. 4G-TGF-a┬1.tif]

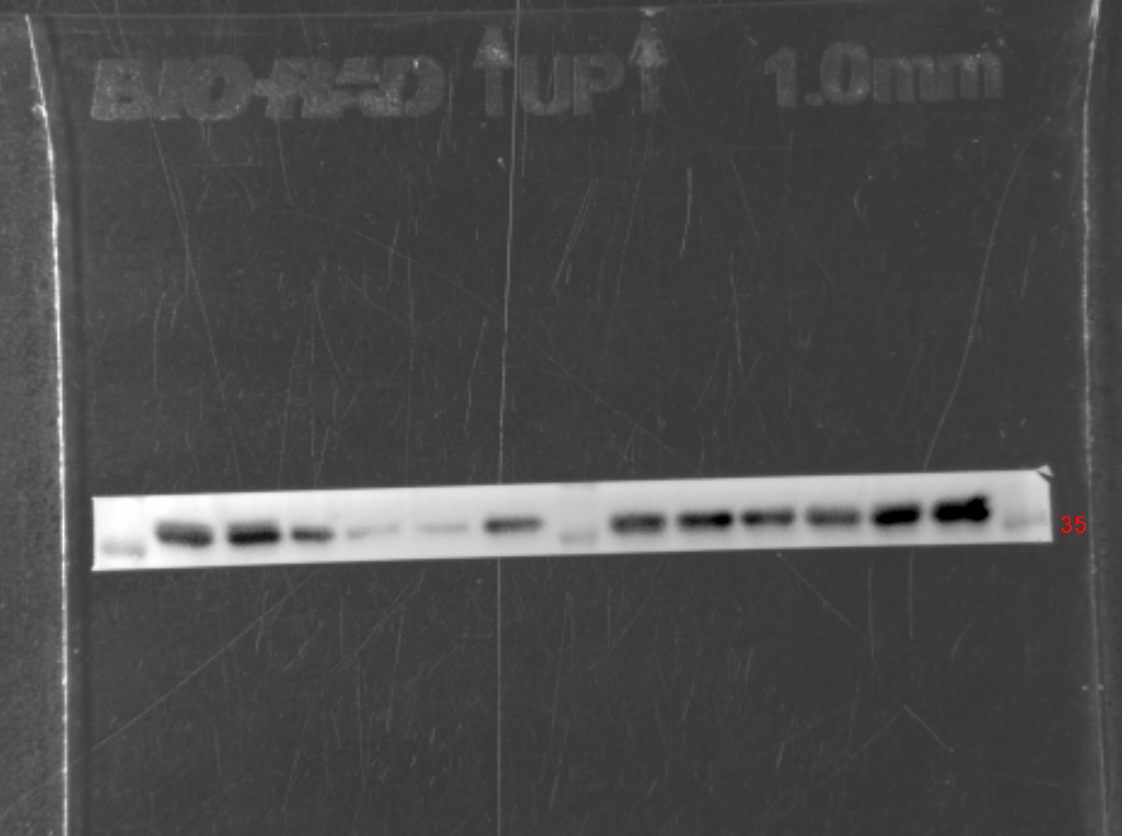

Supplement: Supplementary file 1 — Additional file 1. [file 12891_2021_4712_MOESM1_ESM.zip › merge/Fig. 5A-GAPDH.tif]

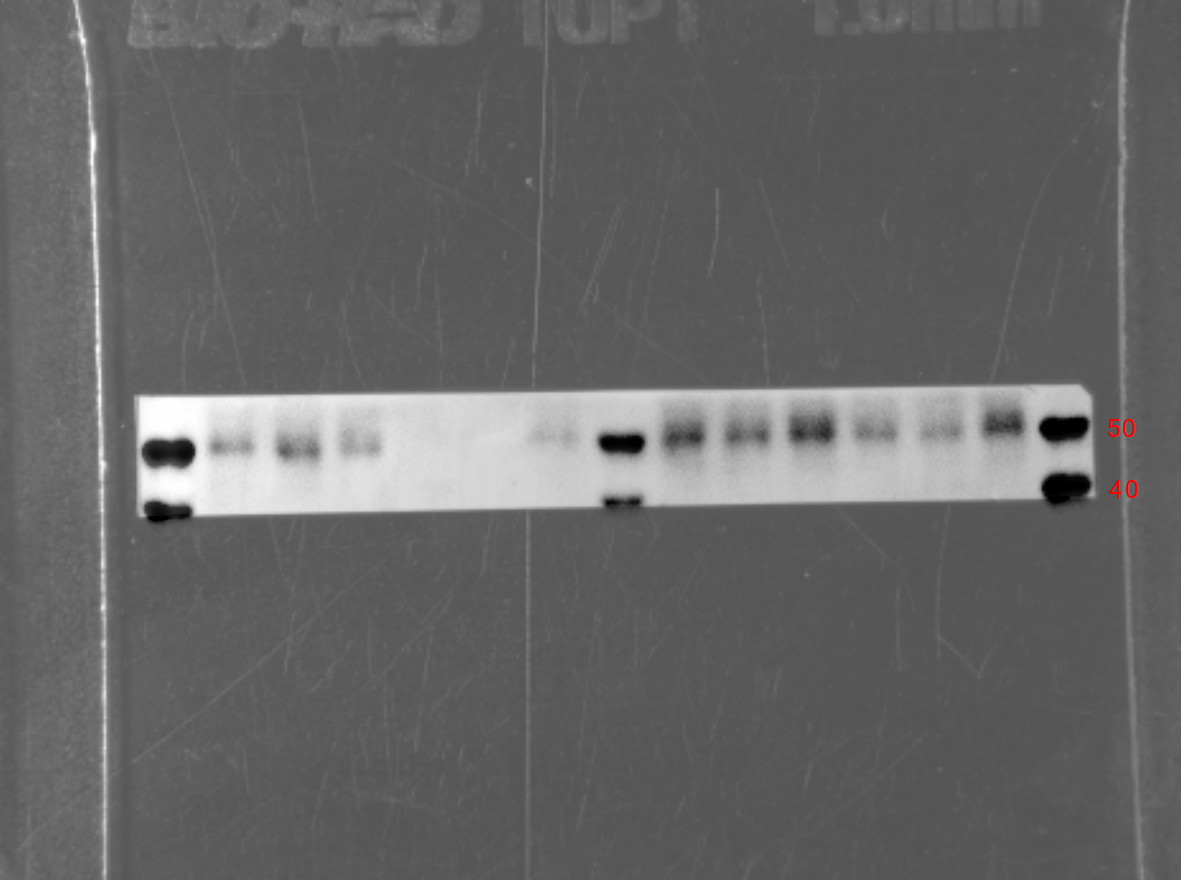

Supplement: Supplementary file 1 — Additional file 1. [file 12891_2021_4712_MOESM1_ESM.zip › merge/Fig. 5A-TGF-a┬1.tif]

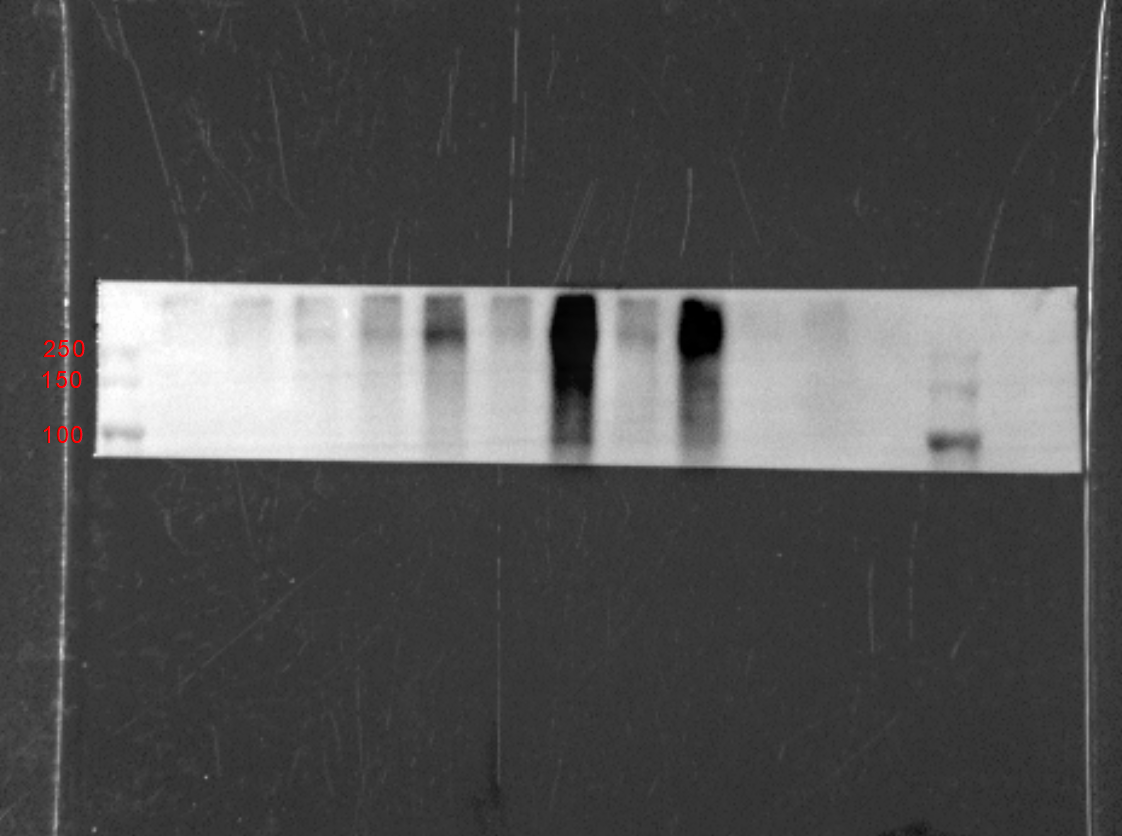

Supplement: Supplementary file 1 — Additional file 1. [file 12891_2021_4712_MOESM1_ESM.zip › merge/Fig. 5B-FN1.tif]

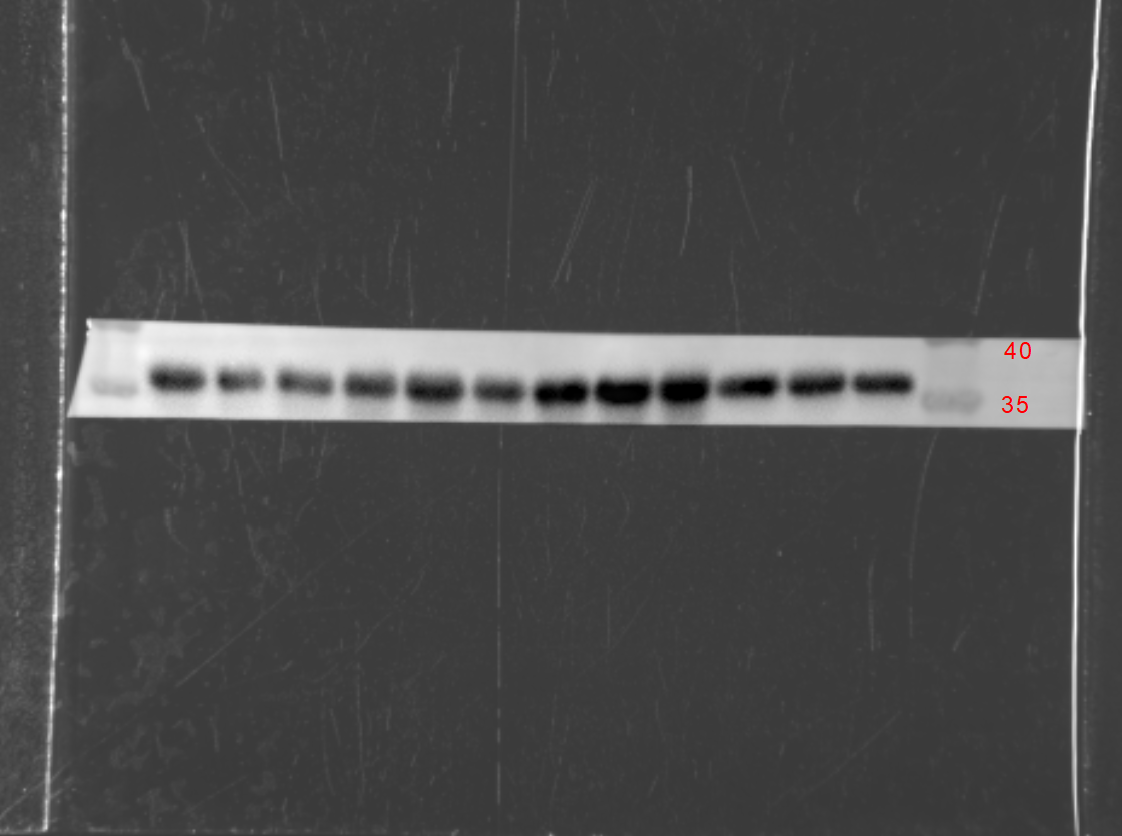

Supplement: Supplementary file 1 — Additional file 1. [file 12891_2021_4712_MOESM1_ESM.zip › merge/Fig. 5B-GAPDH.tif]

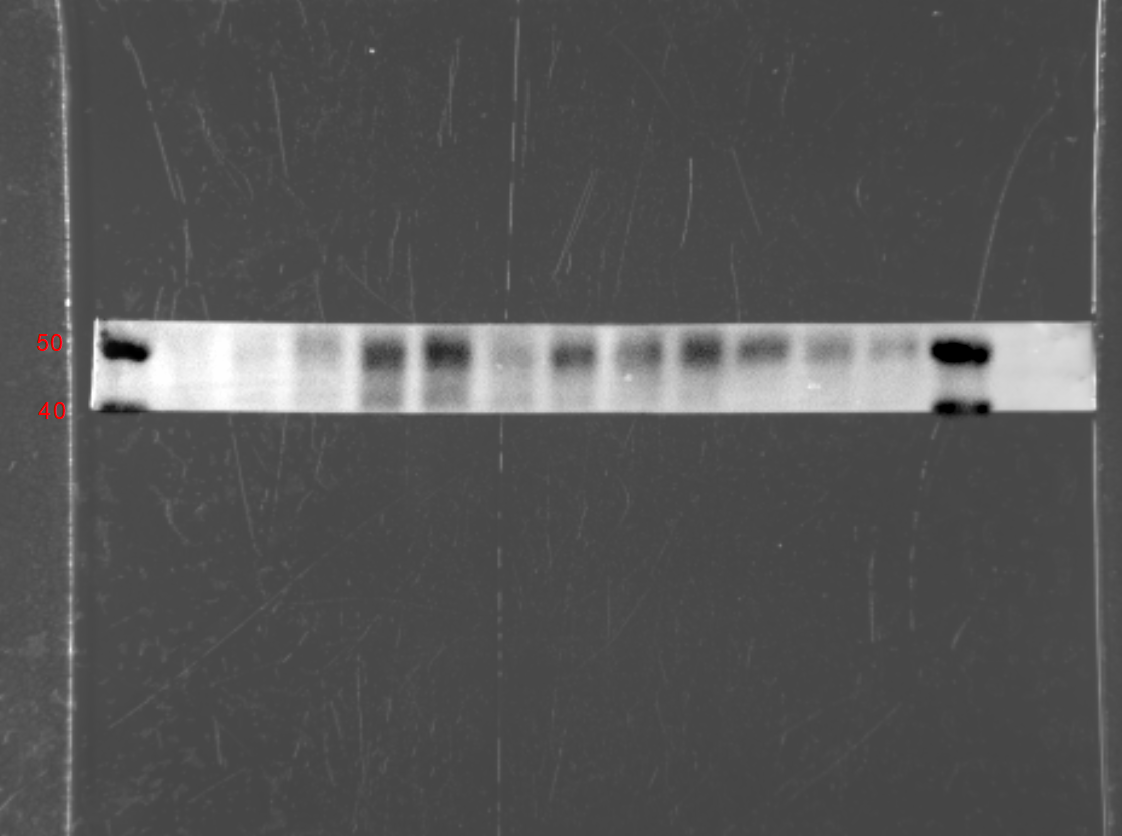

Supplement: Supplementary file 1 — Additional file 1. [file 12891_2021_4712_MOESM1_ESM.zip › merge/Fig. 5B-TGF-a┬1.tif]

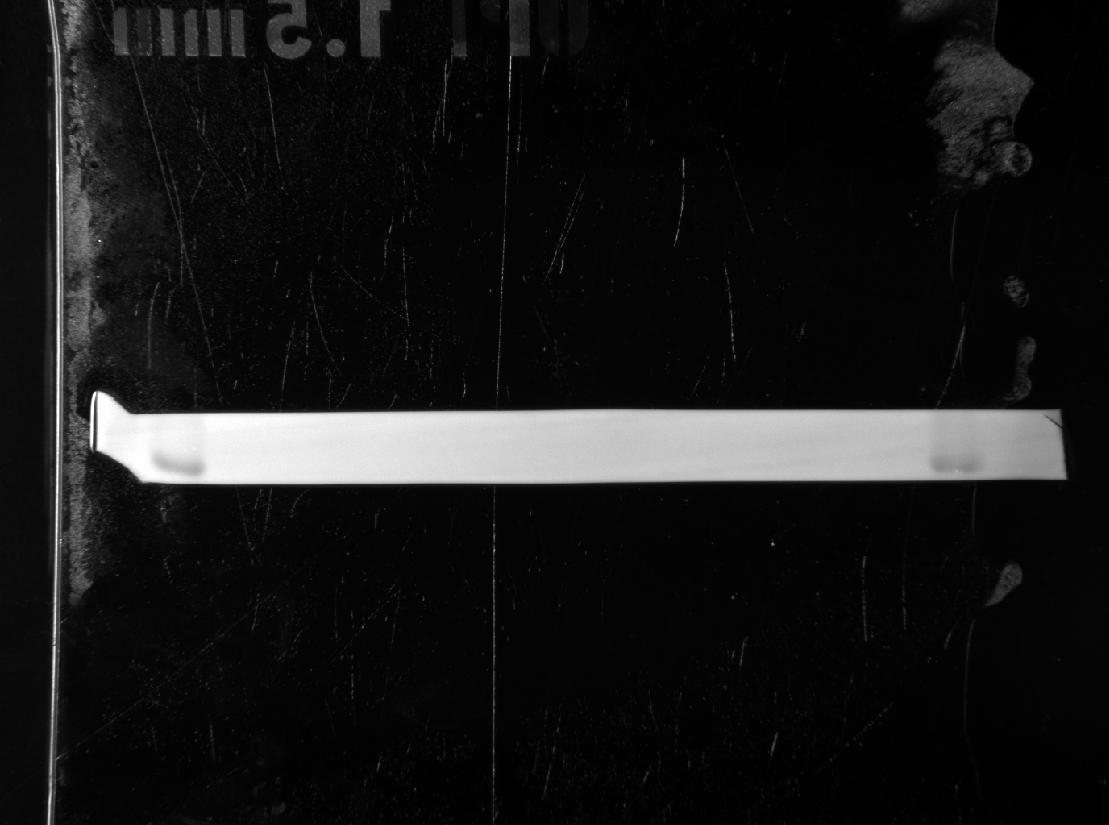

Supplement: Supplementary file 1 — Additional file 1. [file 12891_2021_4712_MOESM1_ESM.zip › merge/Fig. 5C-ERK1-2-image.tif]

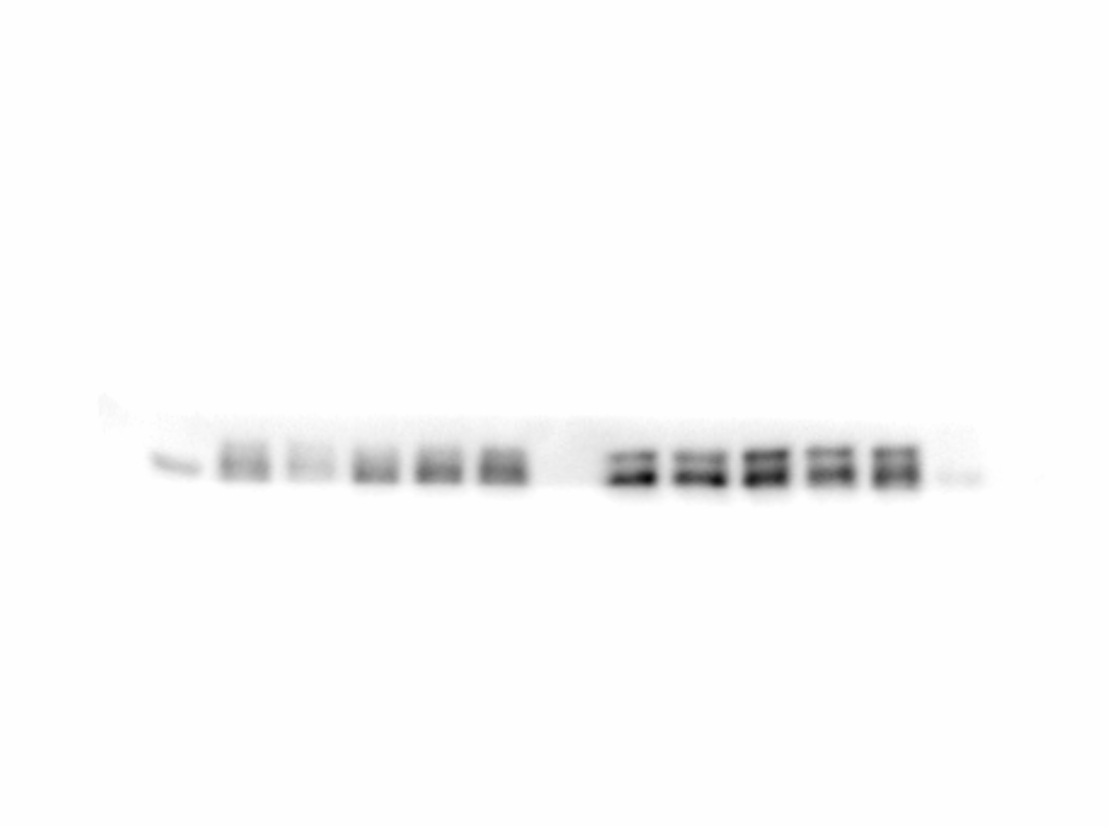

Supplement: Supplementary file 1 — Additional file 1. [file 12891_2021_4712_MOESM1_ESM.zip › merge/Fig. 5C-ERK1-2.tif]

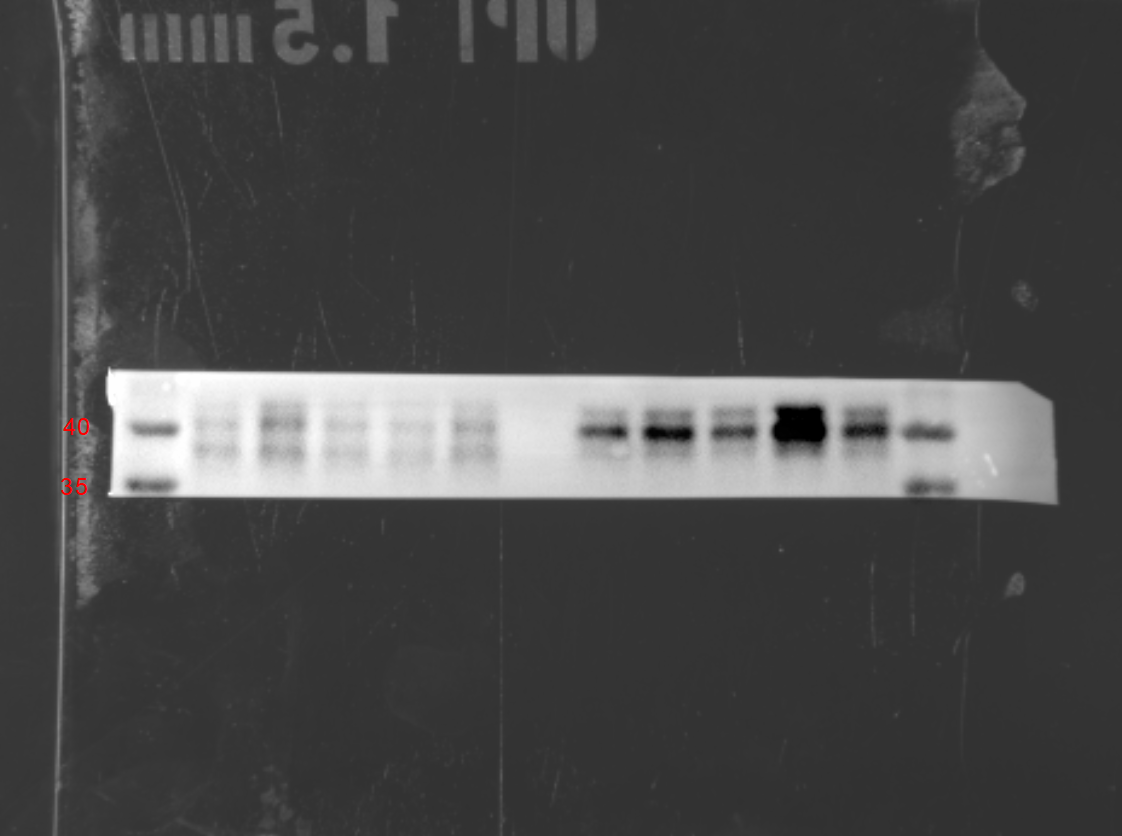

Supplement: Supplementary file 1 — Additional file 1. [file 12891_2021_4712_MOESM1_ESM.zip › merge/Fig. 5C-p-ERK1-2.tif]
